# Identification of genes involved in chicken follicle selection by ONT sequencing on granulosa cells

**DOI:** 10.3389/fgene.2022.1090603

**Published:** 2023-01-12

**Authors:** Dandan Li, Conghao Zhong, Yi Sun, Li Kang, Yunliang Jiang

**Affiliations:** ^1^ Shandong Provincial Key Laboratory of Animal Biotechnology and Disease Control and Prevention, College of Animal Science and Veterinary Medicine, Shandong Agricultural University, Tai’an, China; ^2^ College of Animal Science and Technology, China Agricultural University, Beijing, China

**Keywords:** chicken, follicle selection, Dhcr7, granulosa cells, alternative splicing

## Abstract

In chickens, follicle selection is an important process affecting laying traits, which is characterized by the differentiation of granulosa cells and the synthesis of progesterone by granulosa cells from hierarchical follicles. By using Oxford Nanopore Technologies (ONT) approach, we compared the transcriptomes of granulosa cells between pre-hierarchical (Pre-GCs) and hierarchical follicles (Post-GCs) to identify genes underlying chicken follicle selection. A total of 2,436 differentially expressed genes (DEGs), 3,852 differentially expressed transcripts (DETs) and 925 differentially expressed lncRNA transcripts were identified between chicken Pre-GCs and Post-GCs. For all of the significant DETs, the alternative 3′splice sites (A3) accounted for a maximum of 23.74% of all alternative splicing events. Three DETs of the 7-dehydrocholesterol reductase gene (*DHCR7*) named as T1, T3, and T4, differing in 5′untranslated regions (UTRs), increased in Post-GCs with different folds (T1: 1.83, T3: 2.42, T4: 5.06). The expression of the three *DHCR7* transcripts was upregulated by estrogen in a dose-dependent manner, while was downregulated by bone morphogenetic protein 15 (BMP15) and transforming growth factor-beta 1 (TGF-β1). Follicle-stimulating hormone (FSH) and bone morphogenetic protein 4 (BMP4) promoted the expression of the three *DHCR7* transcripts in Pre-GCs at lower concentrations, while repressed their expression at higher concentrations. The data from this study may provide a reference for better understanding of the genetic mechanisms underlying follicle selection in chicken and other poultry species.

## 1 Introduction

Follicles of various sizes exist in the sexually mature chicken ovaries and these can be broadly divided into pre-hierarchical follicles and hierarchical follicles (F1–F5) (Johnson, 2014). During the laying period, follicles will be cyclically recruited into the preovulatory hierarchy from a cohort of small yellow follicles (SYF, 6–8 mm in diameter) approximately once a day, a process termed follicle selection (Johnson and Woods, 2009). Follicle selection determines the order of hierarchical follicles and is an essential step impacting egg-laying performance in chickens.

An increased number of hormones and cytokines that affect follicle selection have been reported in poultry species including chicken. For sex hormones, follicle-stimulating hormone (FSH) plays an important role in regulating follicular development and maturation (Chu et al., 2018). FSH stimulates the expression of P450scc mRNA and progesterone secretion in the granulosa cells of SYF (Hernandez and Bahr, 2003). Progesterone stimulates the production of fibronectin in chicken granulosa cells and affects oviduct function (Asem et al., 1994). Estrogen affects the hierarchical development of chicken follicles, regulates the proliferation and differentiation of follicular cells (Guo et al., 2018) and also enhances the sensitivity of follicles to luteinizing hormone, thereby increasing progesterone expression in granulosa cells (Caicedo Rivas et al., 2016). Transforming growth factor-beta (TGF-β) is reported to affect the production of steroid hormones, regulate the expression of gonadotropin receptors and stimulate cell proliferation and differentiation (Budi et al., 2017). Bone morphogenetic protein 4 (BMP4) can promote the production of steroid hormones (Yao et al., 2020) and the expression of follicle-stimulating hormone receptors in Pre-GCs, and the differentiation of granulosa cells (Kim et al., 2013). BMP15 is expressed in chicken oocytes and has an inhibitory effect on FSH-induced proliferation of granulosa cells as well as on progesterone production (Elis et al., 2007).

As an important lipid molecule, cholesterol is an essential component of cell membranes for cells to maintain their normal physiological functions. Cholesterol is also a vital precursor for the production of steroid hormones such as estrogen and progesterone, and of bile acids and lipoproteins. Among the enzymes involved in cholesterol synthesis, the 7-dehydrocholesterol reductase (DHCR7) is a key rate-limiting enzyme that removes the C (7–8) double bond in the B ring of sterols and catalyzes the conversion of the 7-dehydrocholesterol to cholesterol. In the synthesis of ovarian steroid hormones from cholesterol, six carbons are first excised from the side chain of cholesterol to produce pregnenolone, which is then catalyzed by 3β-hydroxysteroid dehydrogenase to produce progesterone. Hydroxylation of the 17th carbon of progesterone and cleavage of the side chain of the resulting glucocorticoid can produce androgen; androgen then forms estrogen through aromatization of the A ring. Laying hens need about 300 mg of cholesterol *per* day to lay eggs, of which about 200 mg is deposited in the yolk and the rest will be discharged into the intestine and converted into steroids and vitamin D to support the nutrition requirements (Naber, 1983). In the mammalian ovaries, *de novo* synthesized cholesterol in the granulosa cells of preovulatory follicle is extremely important for the synthesis of progesterone and the release of mature follicles (Sharpe and Brown, 2013).

The synthesis of steroid hormones such as progesterone and estrogen in chicken ovaries is closely related to follicle selection. As a substrate for steroid hormones synthesis, cholesterol may affect follicle selection by influencing steroid hormone synthesis; however, the source of cholesterol and its effect on follicle selection in the ovaries and follicles of poultry species like chicken has not been reported. In the chicken ovarian follicles, the morphology and function of granulosa cells changed significantly before and after follicle selection. The granulosa cells from pre-hierarchical follicles (Pre-GCs) are undifferentiated and can only convert a small amount of cholesterol into progesterone; after follicle selection, the granulosa cells from hierarchical follicles (Post-GCs) respond to FSH and change into a single layer of closely spaced risers that secrete large amounts of progesterone (Onagbesan et al., 2009). To uncover the molecular mechanisms of follicle selection in hens, in this study, we identified the differentially expressed genes and transcripts between chicken Pre-GCs and Post-GCs by using Oxford Nanopore Technologies (ONT) sequencing and found that *DHCR7* was significantly increased. We further analyzed the effects of hormones and cytokines that are related to follicle selection on *DHCR7* expression in chicken Pre-GCs, and analyzed the regulatory mechanisms of its expression to help reveal the function of cholesterol in chicken follicle selection.

## 2 Materials and methods

### 2.1 Animals and sample collection

Hy-Line brown laying hens aged 35 weeks, which had been laying regularly for at least 1 month, were used in this study. The hens were housed individually in laying batteries under standard conditions, with free access to feed and water, under a photoperiod of 16 h light and 8 h dark. The experimental hens were slaughtered by cervical dislocation immediately, and ovaries including all sized follicles were removed from the body and pre-hierarchical follicles and hierarchical follicles were separately placed in ice-cold saline. The birds were handled and treated according to the Institutional Animal Care and Use Ethics Committee of Shandong Agricultural University (No. SDAUA-2022-36). This study was performed according to the Guidelines for Experimental Animals of the Ministry of Science and Technology of China.

### 2.2 Culture and treatment of Pre-GCs and Post-GCs

For chicken follicles, egg yolk was carefully squeezed out with tweezers and washed with phosphate-buffered saline. Pre-GCs were dispersed by treatment with 1% collagenase II (MP Biomedicals, Santa Ana, CA, United States) at 37°C for 7 min with gentle agitation in a beaker. Post-GCs were dispersed by treatment with .25% Trypsin-EDTA (Gibco, Camarillo, CA, United States) at 37°C for 15 min with gentle agitation in a beaker. After centrifugation, the granulosa cells were suspended in medium M199 (Gibco, Camarillo, CA, United States) with 5% fetal bovine serum (Biological Industries, Israel) and 1% penicillin/streptomycin (Solarbio, Beijing, China) and subsequently seeded in 24-well culture plates at an appropriate density for 24 h. Then the serum-free medium was used, and the cells were subsequently treated with different concentrations of estradiol (Sigma, St. Louis, MO, United States of America), recombinant human FSH (R&D Systems, Minneapolis, MN), recombinant human BMP4 (R&D Systems, Minneapolis, MN), recombinant human BMP15 (R&D Systems, Minneapolis, MN), recombinant human TGF-β1 (R&D Systems, Minneapolis, MN) and progesterone (Sigma, St. Louis, MO, United States) for an additional 24 h.

### 2.3 RNA extraction and cDNA library construction

For ONT sequencing, total RNA was extracted using a MicroElute Total RNA Kit (Omega, Norcross, GA, United States) from three Pre-GCs groups and three Post-GCs groups from ovarian follicles of eight Hy-Line brown hens. The quantity and purity of the total RNA were evaluated using Nanodrop and agarose gel electrophoresis. Total RNA was enriched for poly(A) mRNA using the NEBNext Poly(A) mRNA Magnetic Isolation Module. Synthesis of cDNA for sequencing was performed according to the strand-switching protocol from Oxford Nanopore Technologies. Briefly, the cDNA-PCR Sequencing kit (Oxford Nanopore Technologies, Oxford, United Kingdom) was used to prepare full-length cDNA libraries from the poly(A) mRNAs. Then the cDNA was amplified by PCR for 13–14 cycles with specific barcoded adapters from the Oxford Nanopore PCR Barcoding kit (Oxford Nanopore Technologies, Oxford, United Kingdom). Finally, the 1D sequencing adapter was ligated to the DNA before loading onto a FLOPRO002 R9.4.1 flow cell in a PromethION sequencer. MinKNOW was used to run the sequencing. The sequencing data were deposited to the Sequence Read Archive (SRA), National Center for Biotechnology Information (NCBI) with accession number PRJNA891942. The above operations were all performed in Wuhan Benagen Technology Co., Ltd.

### 2.4 Preprocessing, alignments and analysis of novel genes and transcripts

The raw data format of Nanopore sequencing downlink data is fast5 format containing all raw sequencing signals. As a base calling software, Guppy software (version5.0.16) (Knyazev et al., 2020) was used to convert fast5 format data to fastq format data, which contains the base information of sequenced reads and its corresponding sequencing quality information. Low quality reads (average read quality score <7) and short-length reads (<50 bp) were filtered by Nanofilt (version 2.7.1) (De Coster et al., 2018). After removal of the low-quality reads, the remaining reads were subjected to identification and classification of full-length transcripts followed by alignment to the chicken reference genome GRCg6a (https://ftp.ncbi.nlm.nih.gov/genomes/all/GCF/000/002/315/GCF_000002315.6_GRCg6a/) with the aid of the Pychopper v2.4.0 (-Q 7, -z 50) and Pinfish v0.1.0 under default settings, respectively. The abundance of genome-matched transcripts was calculated and normalized as Per Kilobase of exon model per Million mapped reads (TPM) with salmon 1.4.0 (Patro et al., 2017).

### 2.5 Alternative splicing (AS) and transcript factor (TF) analysis

The AS type present for each sample was obtained by SUPPA2 (https://github.com/comprna/SUPPA; parameter: -f ioe-e SE SS MX RI FL) software (Trincado et al., 2018). The software first calculates the psi of each AS event in each group, and then determines whether the AS event is significantly different by examining the difference in psi between groups. Genes with significant differences in psi between two groups at *p* < .05 were deemed to be differentially spliced. AnimalTFDB 3.0 was used to identify TF (Hu et al., 2019).

### 2.6 Analysis of differentially expressed genes, transcripts and lncRNA transcripts

The statistically significant differentially expressed genes (DEGs) and differentially expressed transcripts (DETs) were obtained by an adjusted *p*-value threshold of <.05 and |log_2_ (fold change)| >1 using the DESeq2 software. R package clusterprofiler was used to perform GO functional enrichment and KEGG pathway analyses on DEGs and DETs. GO analysis covers three domains: cellular component (CC), molecular function (MF) and biological process (BP). CPC2 (Coding Potential Calculator 2) (Kong et al., 2007), CNCI (Coding-Non-Coding Index) (Sun et al., 2013) and Pfam (Finn et al., 2014) were used to identify and classify lncRNA. Significantly DE lncRNA transcripts in chicken Pre-GCs and Post-GCs were obtained by an adjusted *p*-value threshold of <.05 and |log_2_ (fold change)| >1.

### 2.7 Rapid amplification of cDNA ends (RACE)

As ONT sequencing revealed four DE transcripts of chicken *DHCR7* gene, differing in 5′untranslated regions (UTRs), in Post-GCs and Pre-GCs, we determined them *via* the 5′RACE method using the SMARTer RACE 5′/3′Kit (TaKaRa, Dalian, China). RACE PCR was performed with the universal Primer Mix primer (UPM-L, UPM-S) and the *DHCR7*-specific primer (*DHCR7*-5GSP1) (Table 1). All PCR amplifications were performed using TransStart FastPfu DNA polymerase (TransGen, Beijing, China). The total reaction volume was 20 μL, containing 1.6 µL of 2.5 mM dNTPs, .4 µL of forward/reverse primer, .4 µL of *TransStart FastPfu* DNA polymerase, 4 µL of 5*×TransStart FastPfu* buffer, 12.2 µL nuclease-free water and 1 µL cDNA. After an initial 2 min 95°C denaturation, samples were cycled 35 times through a denaturation at 95°C for 20 s, annealing for 30 s, and extension at 72°C for 1 min, followed by a final 7 min extension at 72°C.The resulting PCR products were cloned into the pMD19-T vector (TaKaRa, Dalian, China) and sequenced by BGI TECH SOLUTIONS (BEIJING LIUHE) Co., Ltd.

**TABLE 1:** Primers used in this study.

Genes	Strand	Sequence (5′-3′)	Annealing temperature (°C)
*DHCR7*.T1	F	AGAGGAGGTGAGAGACGC	52
	R	ATGCTCTTCCCCACTGTG	
*DHCR7*.T3	F	CTGAGATAAGCGGATCAG	52
	R	TCTACCTCCCATGCTCTT	
*DHCR7*.T4	F	CAGGGTGAGAGTCGGAGT	52
	R	CCC​CAT​AAG​CAA​GTT​GAT​G	
*GAPDH*	F	GAG​GGT​AGT​GAA​GGC​TGC​TG	52
	R	CAC​AAC​ACG​GTT​GCT​GTA​TC	
*DHCR7*-5GSP1	F	CAC​CTG​GAA​AGC​AAC​CCA​AGC​AG	64
UPM-L	R	CTA​ATA​CGA​CTC​ACT​ATA​GGG​CAA​GCA​GTG​GTA​TCA​ACG​CAG​AGT	
UPM-S	R	CTA​ATA​CGA​CTC​ACT​ATA​GGG​C	

### 2.8 RNA isolation, reverse transcription, and quantitative real-time polymerase chain reaction (qRT-PCR)

For qRT-PCR, total RNA was isolated from granulosa cells using the RNA sample Total RNA Kit (Tiangen Biotech, Beijing, China) and quality-checked by 1% agarose gel electrophoresis and spectrophotometer. Reverse transcription was performed using the Evo M-MLV RT Mix Kit with a gDNA Clean (Accurate Biotechnology Co., Ltd., Hunan, China). The qRT-PCR was performed using SYBR Green Premix Pro Tap HS qPCR Kit (Accurate Biotechnology Co., Ltd., Hunan, China) on an LightCycler 480 instrument under the following conditions: 95°C for 30 s; 40 cycles of 95°C for 5 s, and 52°C for 30 s; and a final stage 95°C for 1 s, 54°C for 30 s, and 95°C for 1 s. Melting curves were used to confirm the specificity of each product, and the PCR efficiencies were determined by analysis of two folds serial dilutions of cDNA that were designed to detect all the signals in the spanning region. The efficiencies were nearly 100%, and therefore, the 2^−ΔΔCT^ method for calculating the relative gene expression levels was used (Livak and Schmittgen, 2001) and *GAPDH* gene was used as the internal control. Primer sequences used for qRT-PCR for selected genes are shown in Table 1.

### 2.9 Determination of total cholesterol (TCH) in granulosa cells

The contents of TCH in cells were measured by Total cholesterol assay kit (Nanjing Jiancheng, Jiangsu, China) and quantified as mmol/g total protein.

### 2.10 Statistical analysis

All data were presented as the mean ± SEM. Student’s t-test was used to compare the expression levels of the three *DHCR7* transcripts and the total cholesterol in Pre-GCs and Post-GCs. For other statistical analyses, one-way ANOVA was performed and followed by Duncan’s multiple range test by using SPSS software. For both statistical analyses, *p* < .05 was considered as significantly different.

## 3 Results

### 3.1 Sequencing quality and structural analysis

The transcriptomes of six chicken Pre-GCs and Post-GCs samples were obtained by ONT sequencing (Table 2). Identification of full-length sequences in valid sequencing data yielded 2,735,627-5,084,280 numbers of reads with average read quality score greater than 7 and lengths greater than 50 bp, and 2,161,751-4,311,415 full-length sequences (Table 3). The 28,581 transcripts were functionally annotated in eight databases (Table 4).

**TABLE 2:** Information statistics of clean data from three Post-GCs and three Pre-GCs samples.

Sample name	ReadNum	BaseNum	N50	MeanLength	MaxLength
Post-GCs-1	2,735,627	3,168,127,689	1,759	1,158.1	92,148
Post-GCs-2	3,122,494	3,205,033,012	1,575	1,026.4	591,987
Post-GCs-3	4,134,965	4,512,502,398	1,632	1,091.3	149,661
Pre-GCs-1	3,987,427	3,471,469,185	1,291	870.6	339,670
Pre-GCs-2	4,823,788	3,077,260,255	905	637.9	185,684
Pre-GCs-3	5,084,280	3,182,166,087	879	625.9	223,695

**TABLE 3:** Full-length sequence statistics from three Post-GCs and three Pre-GCs samples.

Sample	PassReads	LenFail	QcFail	Primers_found	Rescue	Unusable
Post-GCs-1	2,735,627	0	0	2,161,751	51,152	549,378
Post-GCs-2	3,122,494	0	0	2,479,586	64,235	612,086
Post-GCs-3	4,134,965	0	0	3,284,139	80,296	812,398
Pre-GCs-1	3,987,427	0	0	3,254,976	67,807	700,131
Pre-GCs-2	4,823,788	0	0	4,113,693	84,084	669,721
Pre-GCs-3	5,084,280	0	0	4,311,415	126,361	711,809

**TABLE 4:** Annotated number of transcripts from three Post-GCs and three Pre-GCs samples.

Item	Annotated_Number	300 = <length<1,000	length> = 1,000
All	28,581 (100.00%)	11,528	11,281
Annotation	15,181 (53.12%)	4,985	9,412
Uniprot	13,387 (46.84%)	4,068	8,848
Pfam	11,447 (40.05%)	3,055	8,183
GO	13,106 (45.86%)	3,980	8,662
KEGG	9,688 (33.90%)	2,948	6,373
Pathway	5,433 (19.01%)	1,619	3,616
COG	3,444 (12.05%)	951	2,365
Eggnog	12,121 (42.41%)	3,657	8,037

**FIGURE 1:**
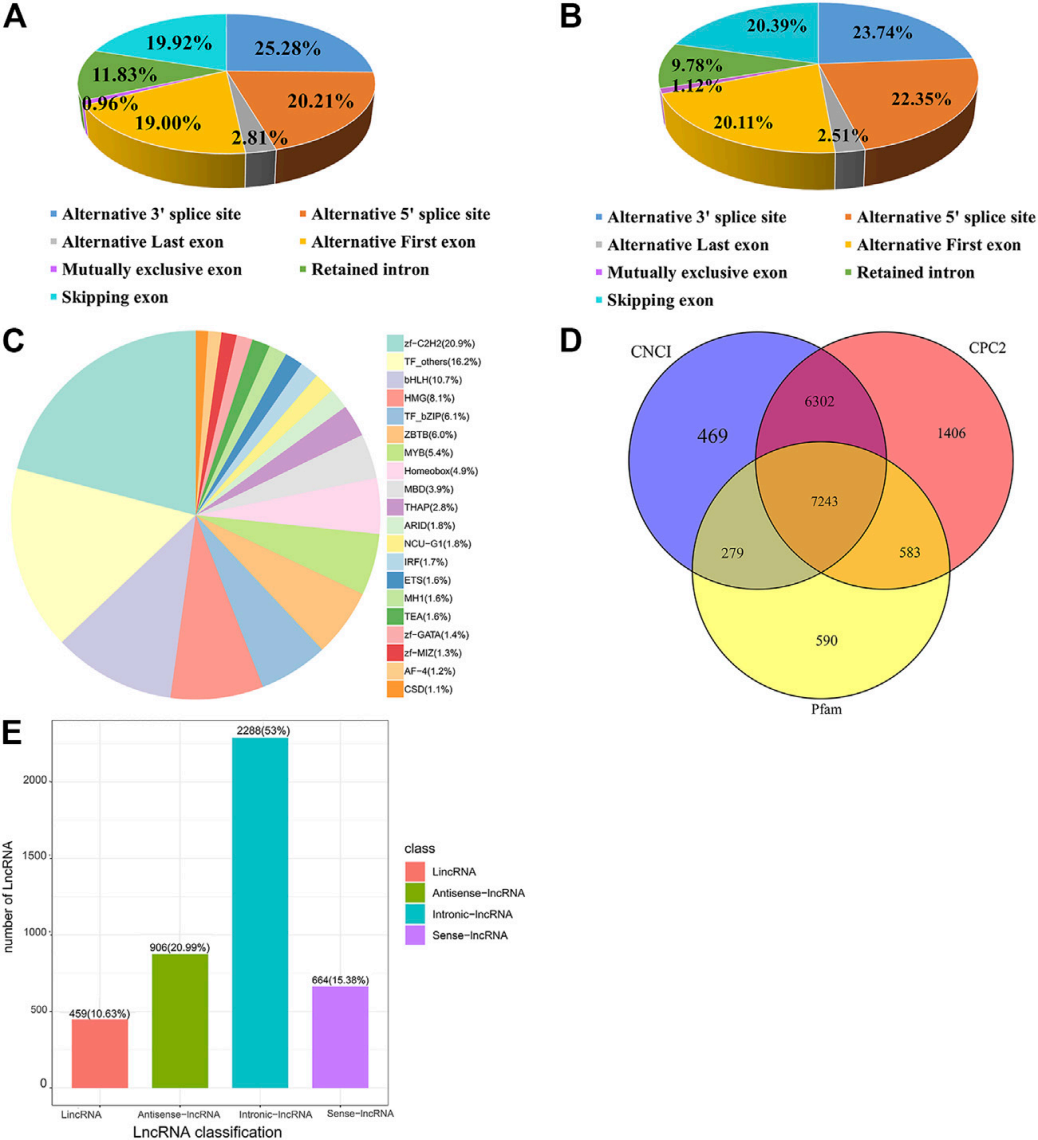
Alternative splicing, transcription factor, and lncRNA analysis of transcripts. **(A)** Percentages of Alternative splicing events. **(B)** The number and proportion of significant differentially expressed alternative splicing events. **(C)** Different families of transcription factors were predicted. **(D)** Venn diagram of lncRNA predicted by the Coding Potential Calculator 2 (CPC2), Coding-Non-Coding Index (CNCI), and pfam methods. **(E)** The classification of lncRNA.

Among the seven alternative splicing types, alternative 3′splice site (A3) accounts for a maximum of 25.28% and mutually exclusive exon (MX) accounts for a minimum of .96% (Figure 1A). The proportion of A3 and MX events is 23.74% and 1.12%, respectively, being the most and the least ones for the significantly DE alternative splicing events (Figure 1B). *Trans*-acting factors (TFs) are DNA-binding proteins that specifically interact with *cis*-acting elements of eukaryotic genes and have an activating or inhibiting effect on gene transcription. In this study, 1,381 TFs were identified and clustered into TF families, most of which were classified into zf-C2H2 family (Figure 1C).

### 3.2 Differentially expressed lncRNA transcripts between chicken Pre-GCs and Post-GCs

By full-length transcriptome sequencing, 7,243 lncRNA were obtained (Figure 1D), of which 459 (10.6%) were lincRNA, 906 (21%) were antisense-lncRNA, 2,288 (53%) were intronic-lncRNA, and 664 (15.4%) were sense-lncRNA (Figure 1E). A total of 925 DE lncRNA transcripts (315 upregulated and 610 downregulated) were identified in Post-GCs. The greatest fold change in lncRNA expression was LOC107053110. t4 (upregulated) and *WSB1*. t10 (downregulated), respectively; and top 50 upregulated differentially expressed lncRNA transcripts and downregulated differentially expressed lncRNA transcripts in Post-GCs are shown in Supplementary Tables S1, S2, respectively.

### 3.3 Differentially expressed mRNA transcripts between chicken Pre-GCs and Post-GCs

**FIGURE 2:**
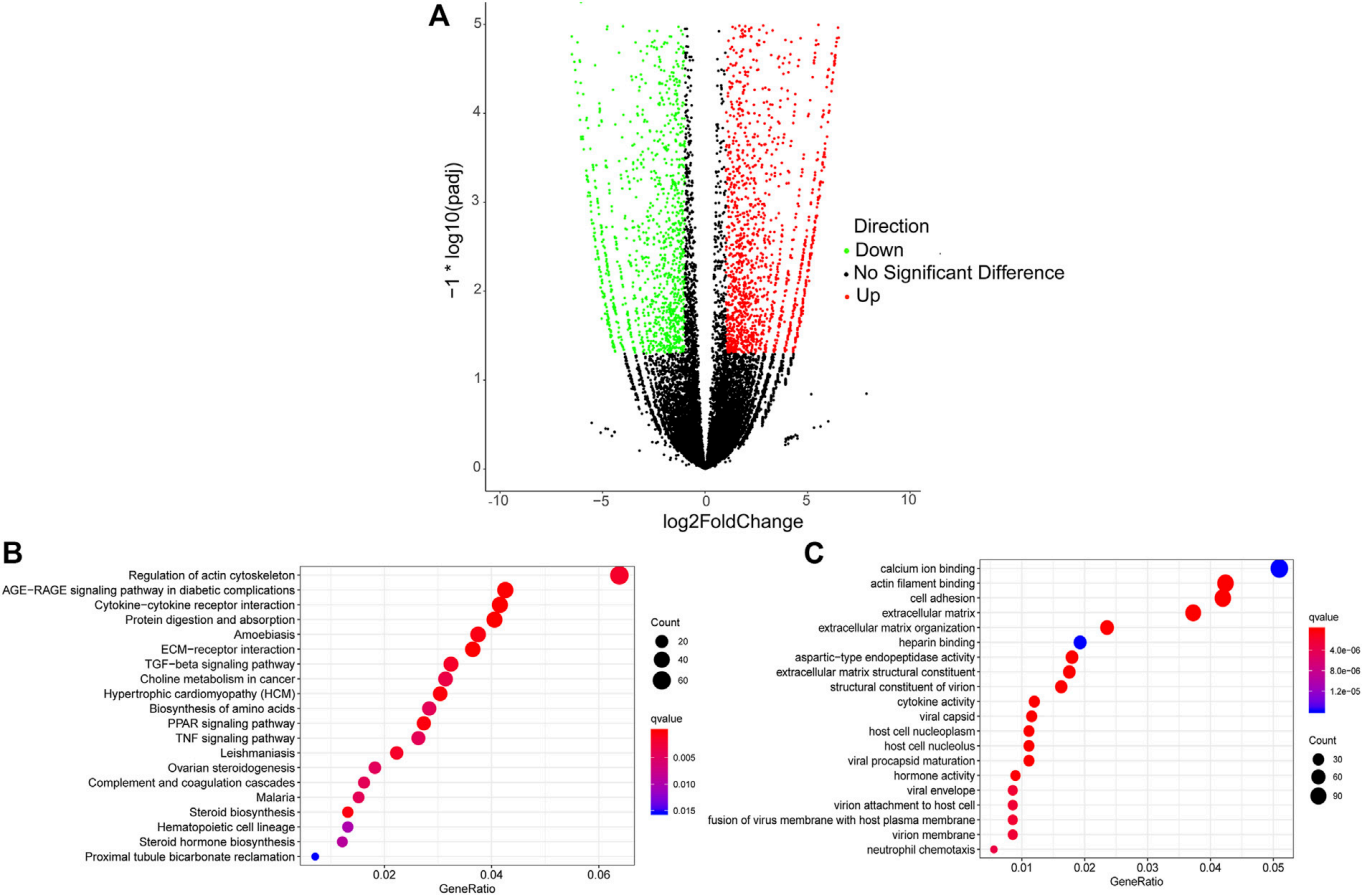
Analysis of differentially expressed transcripts (DETs) between Pre-GCs and Post-GCs in chickens. **(A)** Differentially expressed transcripts between Pre-GCS and Post-GCS in chickens. **(B)** The KEGG analysis diagram of the differentially expressed transcripts. **(C)** The GO analysis diagram of the differentially expressed transcripts.

**FIGURE 3:**
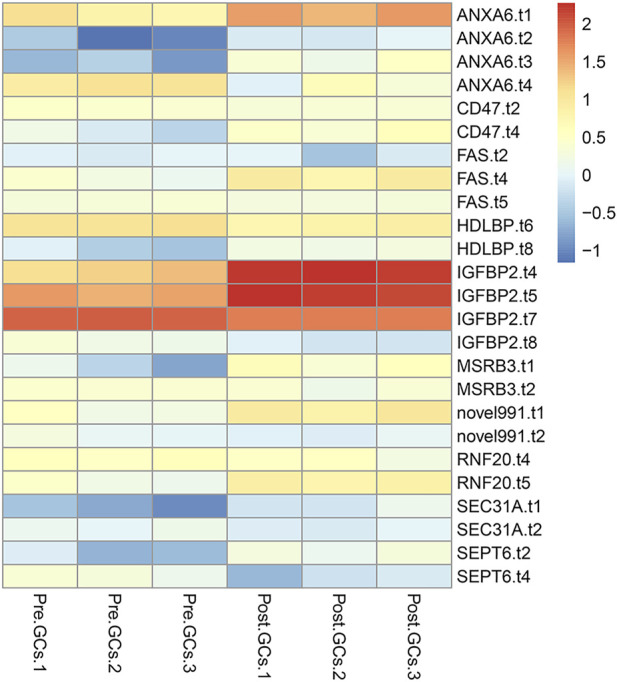
Different transcripts of the same gene displayed different expression patterns in Post-GCs. Only part of the genes are listed; see Supplementary Table S3 for the full list.

A total of 3,852 DETs (1,755 upregulated and 2,097 downregulated) were identified between Pre-GCs and Post-GCs at the significant criteria of (|log_2_ (fold change)| >1 and padj <.05) (Figure 2A). KEGG analysis showed that these DETs were mainly enriched in pathways of regulation of actin cytoskeleton and AGE-RAGE signaling pathway in diabetic complications (Figure 2B) and GO analysis showed that most of these DETs were related to the function of calcium ion binding and actin filament binding (Figure 2C). Some of these alternatively spliced DETs displayed different expression patterns in Post-GCs. For instance, *ANXA6*. t1 was upregulated in Post-GCs, while *ANXA6*. t4 was downregulated in Post-GCs (Figure 3; Supplementary Table S3).

### 3.4 Differentially expressed mRNA genes between chicken Pre-GCs and Post-GCs

**FIGURE 4:**
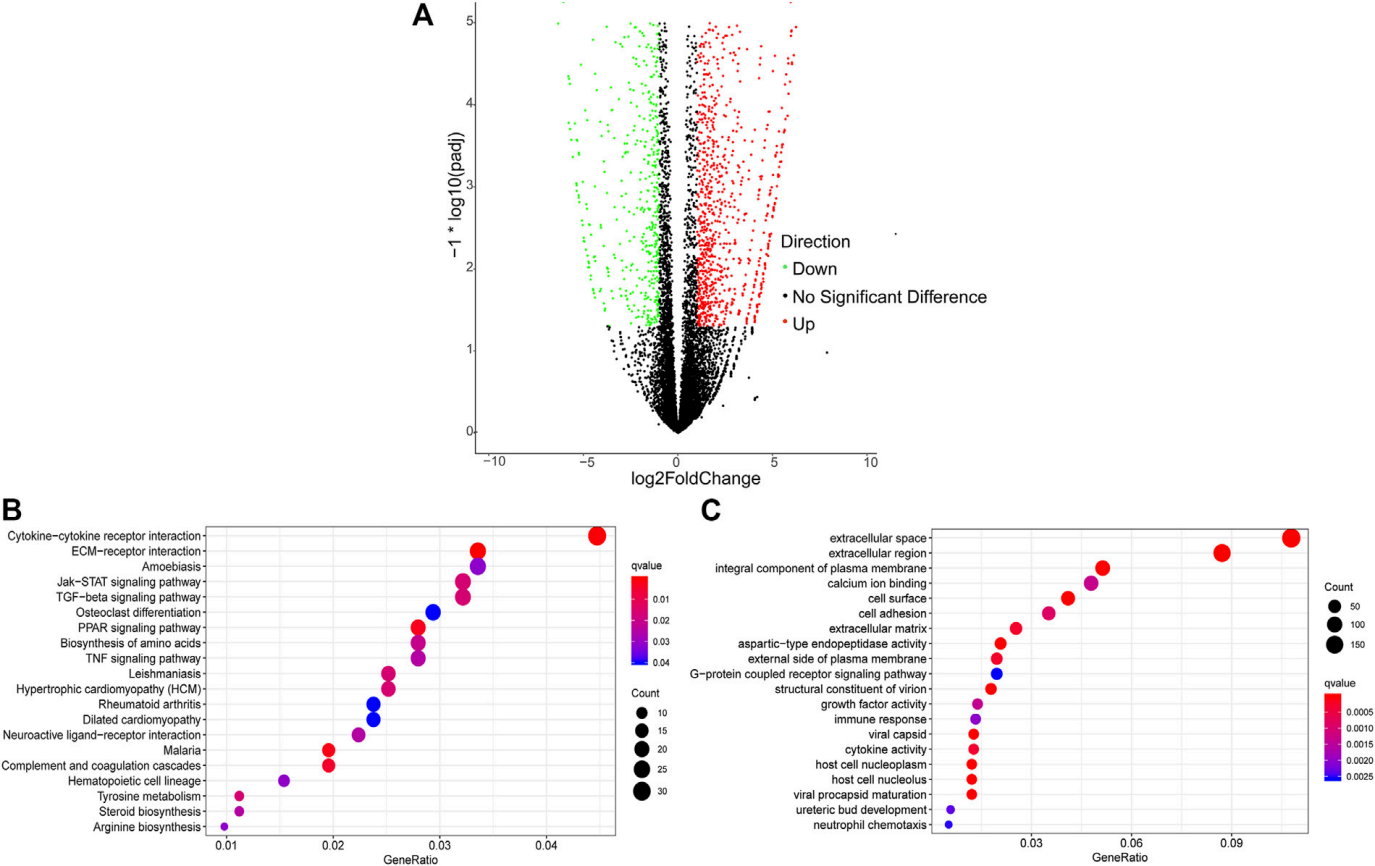
Analysis of differentially expressed genes (DEGs) between Pre-GCs and Post-GCs in chickens. **(A)** Differentially expressed genes between Pre-GCS and Post-GCS in chickens. **(B)** The KEGG analysis diagram of the differentially expressed genes. **(C)** The GO analysis diagram of the differentially expressed genes.

**FIGURE 5:**
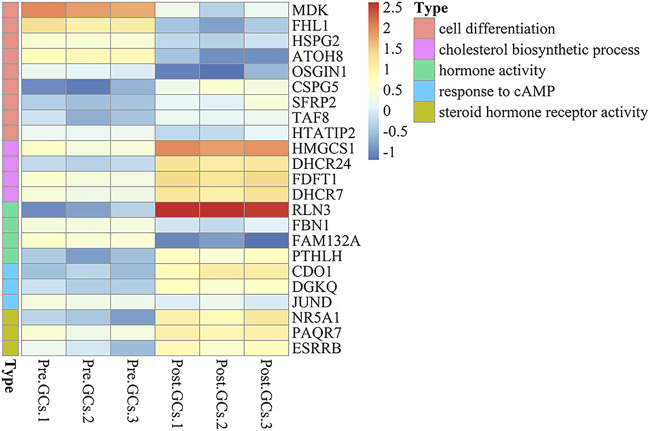
Heatmap analysis of differentially expressed genes (DEGs) related to hormone activity, cell differentiation, cholesterol biosynthetic process, steroid hormone receptor activity and response to cAMP. Only part of the genes are listed; see Supplementary Table S4 for the full list.

A total of 2,436 DEGs (964 upregulated and 1,472 downregulated) were identified between Pre-GCs and Post-GCs (Figure 4A). KEGG analysis showed that these DEGs were mainly enriched in pathways of cytokine−cytokine receptor interaction and ECM−receptor interaction (Figure 4B) and GO analysis showed that most of these DEGs were related to the function of extracellular space and extracellular region (Figure 4C). The expression of DEGs related to follicle development, and those enriched in hormone activity, cell differentiation, cholesterol biosynthetic process, steroid hormone receptor activity and response to cyclic adenosine monophosphate (cAMP) were shown in Figure 5 and Supplementary Table S4. The top 50 upregulated and downregulated genes in Post-GCs are listed in Supplementary Tables S5, S6, respectively.

### 3.5 Three transcripts of *DHCR7* increased in chicken Post-GCs

**FIGURE 6:**
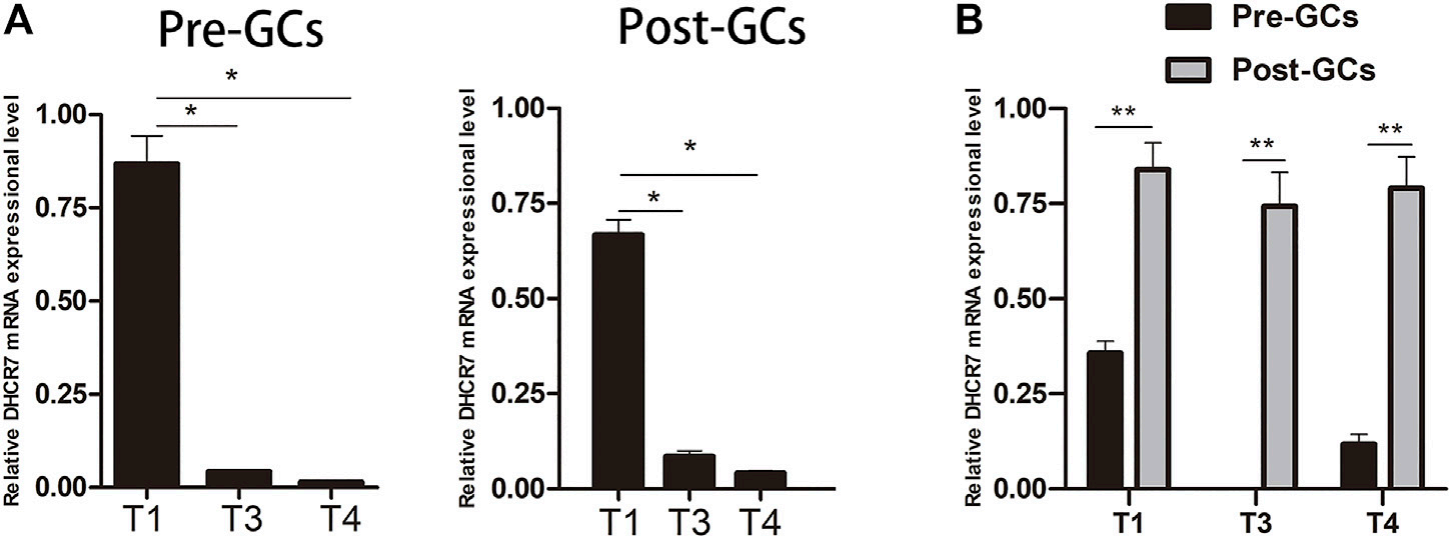
Expression of three *DHCR7* transcripts in chicken Pre- and Post-GCs **(A)** and their comparison **(B)**. T1, T3 and T4 represent the three transcripts of chicken *DHCR7* gene. All data were presented as the means ± SEM. ∗*p* < .05, ∗∗*p* < .01.

ONT sequencing revealed that *DHCR7* was differentially expressed between Pre-GCs and Post-GCs, the expression level of three *DHCR7* transcripts significantly increased in Post-GCs, but with different folds (T1: 1.83, T3: 2.42, T4: 5.06). DHCR7 is enriched in the steroid biosynthesis pathway (Figure 4B) and is involved in cell differentiation and cholesterol biosynthetic process (Supplementary Table S4). Their expression and regulation by sex hormones and cytokines were subsequently examined. By sequence alignment of the three *DHCR7* transcripts, we found that they have the same coding region but with different 5′UTRs. Transcripts T1 and T3 were also detected by 5′RACE technique and sequencing (Supplementary Figure S1). Expression dynamics of T1, T3, and T4 transcripts of *DHCR7* between Pre- and Post-GCs were verified by qRT-PCR (Figure 6; *p* < .05), which were consistent with transcriptome sequencing results.

### 3.6 Total cholesterol content increased in chicken Post-GCs

**FIGURE 7:**
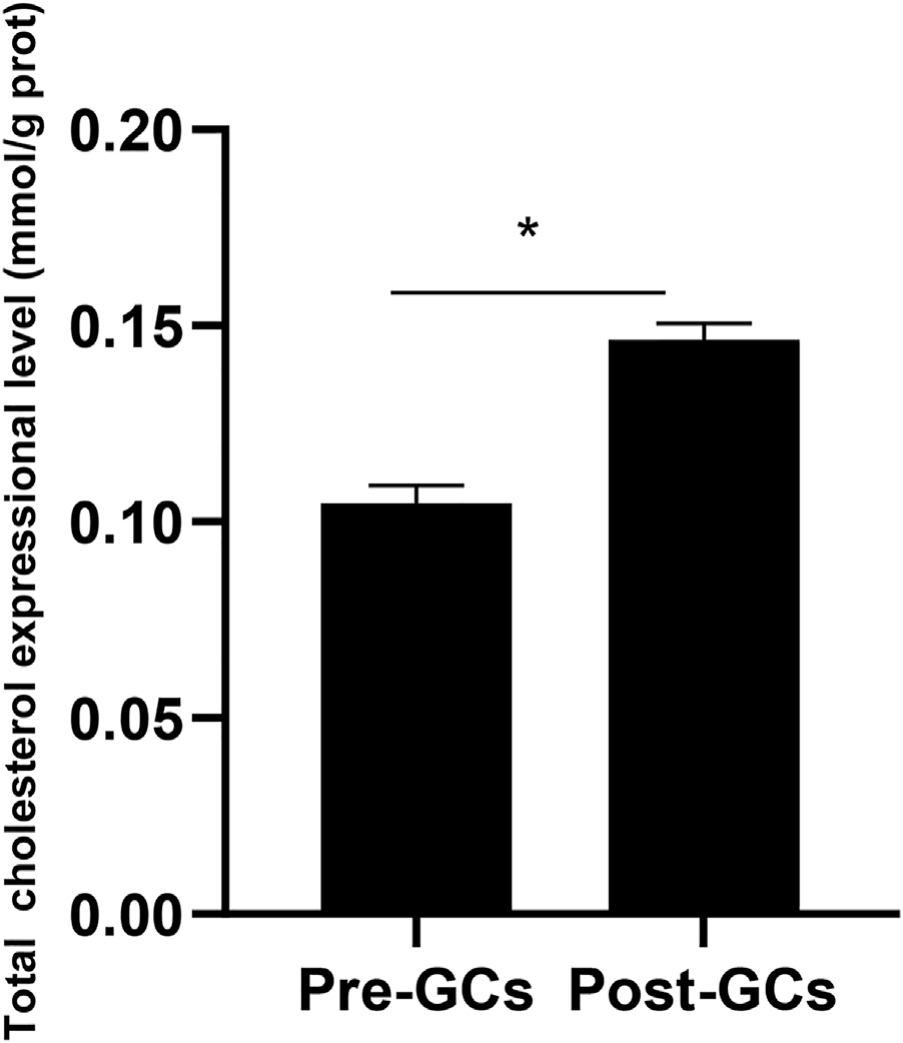
Total cholesterol expression in Pre-GCs and Post-GCs in chickens. All data were presented as the means ± SEM. ∗*p* < .05.

Due to that the expression of *DHCR7* showed an significant upregulation in Post-GCs, and DHCR7 can catalyze the formation of cholesterol from the 7-dehydrocholesterol, we measured the total cholesterol content in chicken Pre-GCs and Post-GCs and found that the total cholesterol content was significantly higher in Post-GCs (Figure 7; *p* < .05). The increased level of cholesterol in Post-GCs is possibly caused by increased expression of three *DHCR7* transcripts. Therefore, the effect of sex hormones and cytokines on the expression of chicken *DHCR7* was further analyzed.

### 3.7 Effect of estrogen, progesterone and FSH on *DHCR7* mRNA expression in chicken Pre-GCs

**FIGURE 8:**
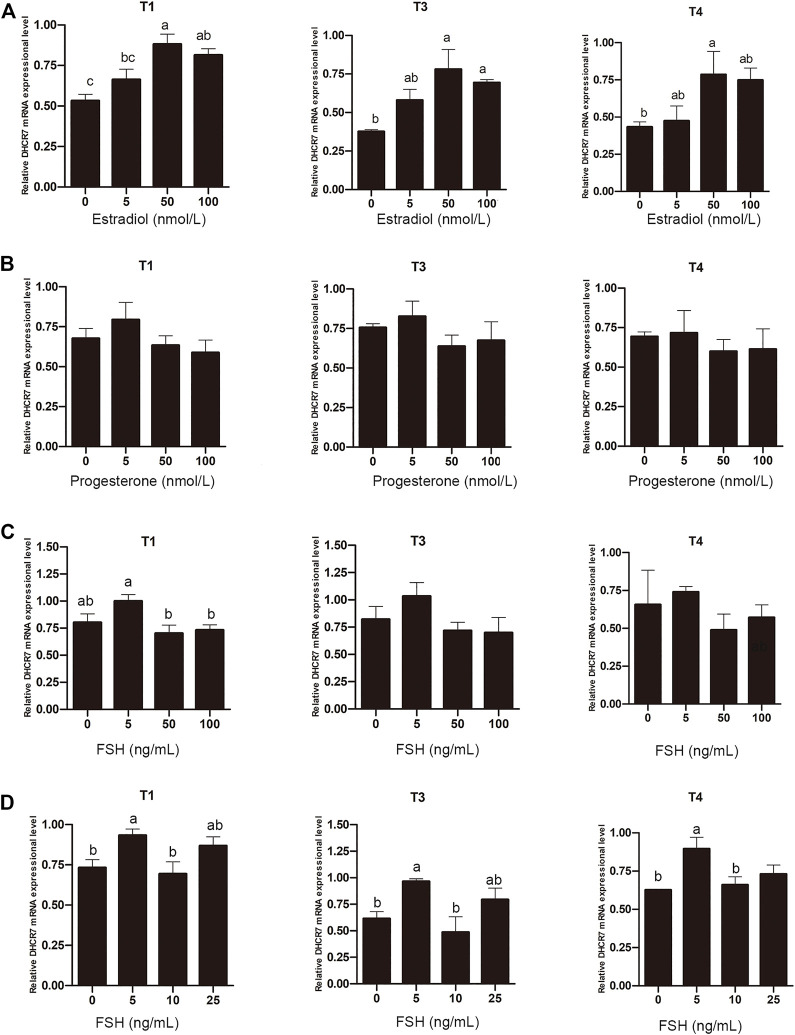
Effect of estradiol **(A)**, progesterone **(B)**, and FSH **(C,D)** on the mRNA expression of three *DHCR7* transcripts in chicken Pre-GCs. T1, T3, and T4 represent the three transcripts of chicken *DHCR7* gene. All data were presented as the means ± SEM. Different letters indicate that the difference was significant (*p* < .05).

Unlike mammals that synthesize estrogen in granulosa cells, sexually mature hens synthesize estrogen in theca cells from pre-hierarchical follicles. To determine whether estrogen is involved in regulating the expression of *DHCR7*, Pre-GCs were treated with 0, 5, 50, and 100 nmol/L estradiol, respectively, and it was found that estradiol significantly promoted the expression of *DHCR7* transcripts T1, T3 and T4 in a dose-dependent manner; the effect was most pronounced at 50 nmol/L (Figure 8A; *p* < .05). Treatment of Pre-GCs with 0, 5, 50, and 100 nmol/L of progesterone revealed no significant effect on the expression of any of the three *DHCR7* transcripts (Figure 8B; *p* > .05). Treatment of Pre-GCs with FSH at low concentrations promoted the expression of the three *DHCR7* transcripts, while at high concentrations repressed their expression (Figure 8C; *p* < .05). Further narrowing the concentration gradient revealed that the expression of the three *DHCR7* transcripts was significantly promoted only at a concentration of 5 ng/mL, and the expression of the three *DHCR7* transcripts was inhibited at a concentration greater than 10 ng/mL (Figure 8D; *p* < .05). These data showed that the effect of estrogen, progesterone and FSH on chicken *DHCR7* expression was different.

### 3.8 Effect of BMP15, TGF-β1 and BMP4 on *DHCR7* mRNA expression in chicken Pre-GCs

**FIGURE 9:**
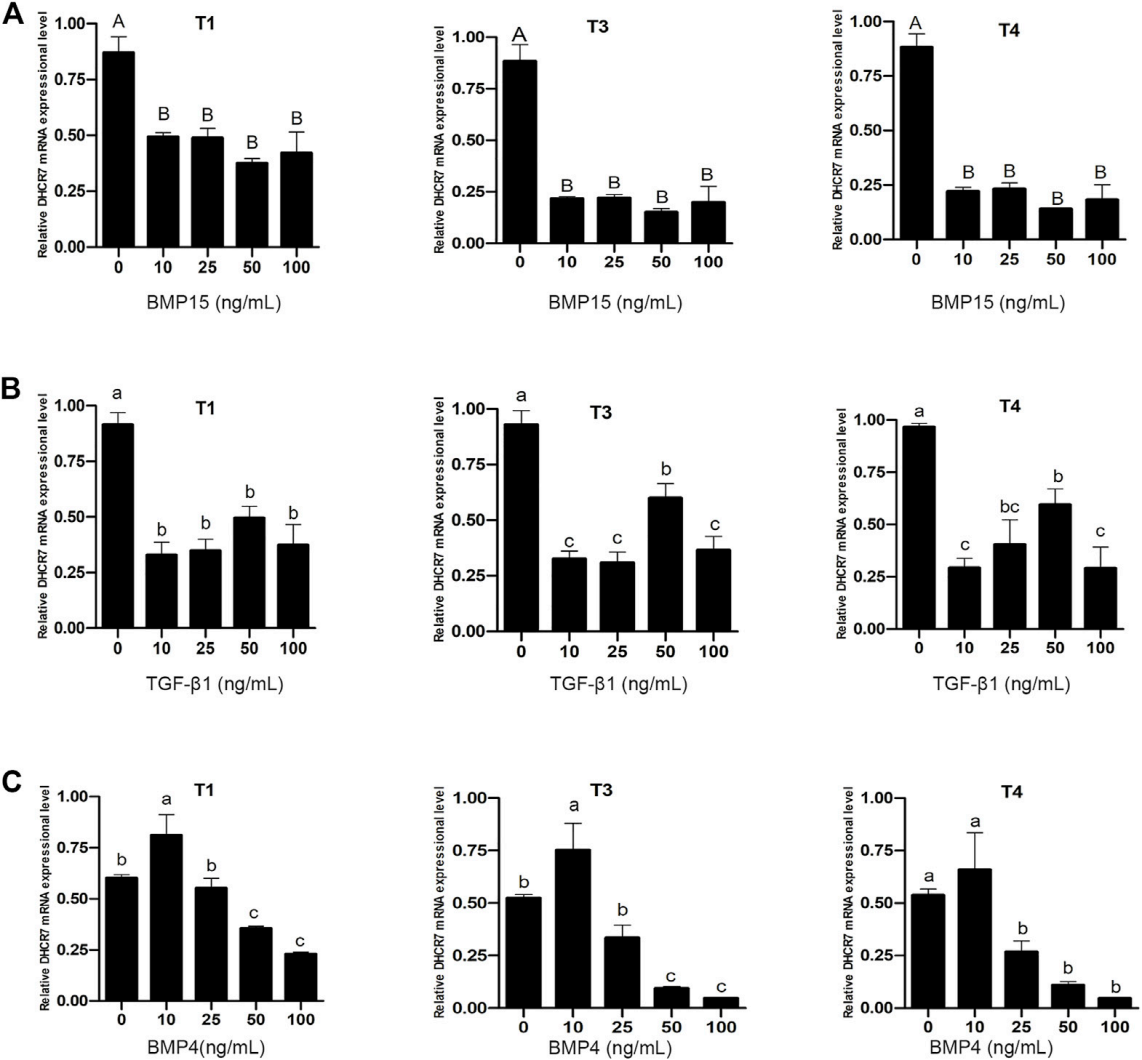
Effect of BMP15 **(A)**, TGF-β1 **(B)** and BMP4 **(C)** on the mRNA expression of three *DHCR7* transcripts in chicken Pre-GCs. T1, T3, and T4 represent the three transcripts of chicken *DHCR7* gene. All data were presented as the means ± SEM. Different letters indicate that the difference was significant: lowercase letters, *p* < .05; uppercase letters, *p* < .01.

Pre-GCs were treated with BMP15, TGF-β1 and BMP4, respectively, at different concentrations of 0, 10, 25, 50, and 100 ng/mL. It was found that both BMP15 (Figure 9A) and TGF-β1 (Figure 9B) inhibited the expression of three *DHCR7* transcripts (*p* < .05). For BMP4, it significantly promoted the expression of the three *DHCR7* transcripts at the concentration of 10 ng/mL, while higher concentrations had a repressive effect on the expression of the three *DHCR7* transcripts (Figure 9C; *p* < .05). The similar effect of BMP15 and TGF-β1, and different effect of BMP4 on chicken *DHCR7* expression is likely due to that different cells (somatic cells vs. oocyte) expressed these cytokines.

## 4 Discussion

Sexually mature hens have approximately 12,000 oocytes in their ovaries, but only a few hundred are able to be selected and develop to maturity and ovulation (Onagbesan et al., 2009). Differences in the efficiency of follicle selection are directly related to egg laying in chickens. However, the mechanism of follicle selection is not yet fully understood. In this study, in order to identify genes underlying chicken follicle selection and understand the genetic and physiological mechanisms of follicle selection, for the first time, we compared the transcriptomes of granulosa cells from pre-hierarchical and hierarchical follicles using ONT transcriptome sequencing approach. Bioinformatics analysis was performed on the sequencing data, and the regulation of differentially expressed gene *DHCR7* by hormones and some cytokines were investigated.

ONT sequencing is a new generation of nanopore-based single-molecule real-time electrical signal sequencing technology, which, unlike previous RNA sequencing technologies, is a long-read technology that provides full-length transcripts, accurate analysis of alternative splicing, fusion genes and identification of novel isoforms, enabling accurate quantification of expressed transcripts (Li et al., 2021). In this study, thousands of mRNA genes or transcripts and hundreds of lncRNA transcripts were found to be differentially expressed and alternatively spliced pattern of some DEGs were revealed between Pre-GCs and Post-GCs. Especially, some DEGs were predicted to be involved in hormones activity, cell differentiation, cholesterol biosynthetic process, steroid hormone receptors activity and response to cAMP, which are essential for follicle selection in chickens.

Among the DEGs identified, the expression changes of Anti-Mullerian hormone (*AMH*), 24-dehydrocholesterol reductase (*DHCR24*), adrenomedullin 2 (*ADM2*) and Nuclear receptor subfamily 2 group F member 2 (*NR2F2*) in granulosa cells were notable. In chicken, *AMH* is mainly expressed in the granular layer of chicken ovarian follicles, significantly reduced after follicle selection (Johnson, 2012) and inhibits the development of Pre-GCs in laying hens (Huang et al., 2021). The sequencing data from this study showed that the mRNA expression of *AMH* was significantly lower in Post-GCs (Supplementary Table S4), suggesting that *AMH* negatively regulates the differentiation of granulosa cells during chicken follicle selection. By catalyzing the reduction of the C-24 double bond of sterol intermediates during cholesterol biosynthesis, *DHCR24* is involved in cell growth, senescence and cellular response to oncogenic and oxidative stress (Daimiel et al., 2013). Knockdown of *DHCR24* inhibited the metastatic ability of endometrial cancer cells and upregulated progesterone receptor expression (Dai et al., 2017). In this study, we found that the mRNA expression of *DHCR24* was significantly higher in Post-GCs (Supplementary Table S4), suggesting an active role in cholesterol biosynthetic process. This is also consistent with the fact that cholesterol level significantly increased in Post-GCs (Figure 7). Follicle selection is regulated by the hypothalamic-pituitary-gonadal axis, and the activity of hormones and their receptors is a prerequisite for the function of the hypothalamic-pituitary-gonadal axis. *ADM2* increases the synthesis and secretion of 17β-estradiol, as well as the expression of steroidogenic factor 1, estrogen receptor α, and enzymes involved in steroidogenesis in equine chorionic gonadotropin treated rat ovaries (Chauhan et al., 2015). In growing rat ovaries, inhibition of *IMD*/*ADM2* signaling results in oocyte atresia and abnormal cell cycle progression in follicular cells (Chang et al., 2011). Our sequencing data showed that the mRNA expression of *ADM2* was lower in Post-GCs (Supplementary Table S4), suggesting that it plays an inhibitory role on follicle growth. *NR2F2* is a master regulator of angiogenesis (Hawkins et al., 2013) and is reported to stimulates progesterone synthesis in porcine granulosa cells (Guo et al., 2020). We found that, in chicken Post-GCs, the mRNA expression of *NR2F2* decreased (Supplementary Table S4). The role of *NR2F2* in chicken follicle selection needs further investigations.

For some DETs, different transcripts of the same gene displayed different expression patterns in Post-GCs. For instance, *ANXA6*. t1 was upregulated in Post-GCs, while *ANXA6*. t4 was downregulated in Post-GCs. Annexin A6 (*ANXA6*) is a calcium-dependent, phospholipid-binding protein found in various cells and tissues. In addition, *ANXA6* participates in cholesterol storage and the control of late endosomal cholesterol levels that modulate cell migration (García-Melero et al., 2016). *ANXA6* is correlated with microtubule-associated protein 1 light chain 3 in cervical cancer and inhibits tumorigenesis through autophagy induction (Sun et al., 2020). *ANXA2*, a gene in the same family as *ANXA6*, is frequently up-regulated in various tumors, such as ovarian cancer. *ANXA2* is associated with folliculogenesis and contributes to follicle development and ovulation in chicken (Zhu et al., 2015). In the follicle, insulin-like growth factor binding protein 2 (*IGFBP2*) is regulated by steroids, FSH, and luteinizing hormone, and there is experimental evidence that steroidogenesis is also negatively co-regulated by *IGFBP2* (Mazerbourg and Monget, 2018). In this study, four *IGFBP2* transcripts are differentially expressed with different expression patterns in Pre- and Post-GCs of chicken follicles. Similar results were also found in the ONT transcriptome sequencing of *Coilia nasuseyes* showing that *ODCs* of *ODC-2*, *ODC-3*, *ODC-4,* and *ODC-5* exhibited upregulation in the hypotonic environment, while *ODC-4* and *ODC-5* exhibited downregulation in the hyperosmotic environment (Gao et al., 2021) and the two transcript isoforms generated from dehydrin 4 (*DHN4*) performed different responses to abiotic stress in *Arabidopsis thaliana* (Lv et al., 2018). We propose that different transcripts of the same gene exhibiting different expression patterns may indicate that they have different functions. Uncovering the functions and regulatory mechanisms of these genes will help to better understand the mechanism of follicle selection.

The precursor mRNA produced by transcription of some genes can produce two or more mRNAs in different ways, which is called alternative splicing. By alternative 5′splice sites, the precursor mRNA of *DHCR7* produces three transcripts of T1, T3, and T4. The 7-dehydrocholesterol reductase encoded by *DHCR7* is a 55 KDa protein with a primary structure of 475 amino acids, which is highly conserved among species (Sharpe and Brown, 2013). In the BLOCH pathway of cholesterol synthesis, DHCR7 converts the 7-dehydrosterols to desmosterol to form cholesterol. In the KANDUTSCH RUSSELL pathway of cholesterol synthesis, DHCR7 catalyzes the formation of cholesterol by the 7-dehydrocholesterol under the action of NADH. DHCR7 affects the Hedgehog signaling pathway (Bijlsma et al., 2006) and the production of vitamin D3 (Prabhu et al., 2016b); it also participates in lipid metabolism, the occurrence of diabetes and the formation of insulin resistance (Li et al., 2009). The interaction of DHCR7 with emopamil binding protein promotes cell growth and differentiation (Prabhu et al., 2016a). Homozygous and heterozygous mutations in human *DHCR7* lead to the occurrence of Smith-Lemli-Opitz syndrome (SLOS), which is manifested by elevated levels of the 7-dehydrocholesterol and little cholesterol synthesis in patients, and symptoms such as limb defects, genital abnormalities, and cognitive impairment (Malgorzata and Nowaczyk, 1998). At present, there are studies on the expression changes of chicken *DHCR7* in liver (Jiang et al., 2021) and adipose precursor cells (Regassa and Kim, 2015), but there is no report on the function of *DHCR7* in chicken follicle selection. In this study, we found that the three *DHCR7* transcripts are higher in Post-GCs and that estrogen up-regulated the expression of three transcripts of *DHCR*7 in a dose-dependent manner, while BMP15 and TGF-β1 inhibited the expression of three transcripts. In hens, Pre-GCs are in an undifferentiated state due to mitogen-activated protein kinases (MAPK) inhibition and can only convert a small amount of cholesterol into progesterone (Johnson and Woods, 2009). As follicle selection proceeds, granulosa cells respond to FSH and differentiate, secreting large amounts of progesterone (Onagbesan et al., 2009). The increased expression of *DHCR7* and *DHCR24* in Post-GCs results in higher level of cholesterol, which provides more substrate for progesterone synthesis. This is consistent with the higher total content of cholesterol in Post-GCs. The regulation of *DHCR7* by estrogen, TGF-β1 and BMP15 may suggest a vital role of *DHCR7* in granulosa cells during chicken follicle selection. One study revealed that the expression of BMP4 increased in Post-GCs compared to Pre-GCs, and BMP4 stimulated the expression of follicle-stimulating hormone receptors in Pre-GCs at the concentration of 10–100 ng/mL when co-treated with FSH for 3 h; however, its promoting effect on progesterone synthesis is only detected at 10 and 25 ng/mL, higher concentration of BMP4 has a trend to decrease progesterone synthesis (Kim et al., 2013). In this study, we found a repressive effect of BMP4 on *DHCR7* mRNA expression at concentrations higher than 10 ng/mL, similar to its effect on progesterone, which is consistent with the role of *DHCR7* in progesterone synthesis, and suggesting that *DHCR7* transcription is tightly regulated by BMP4.

Cytochrome P450 aromatase is the key rate-limiting enzyme for estrogen synthesis and is encoded by *CYP19A1*. In the chicken ovaries, *CYP19A1* is expressed only in theca cells and not in granulosa cells, where estrogen is produced by theca cells of pre-hierarchical follicles and progesterone is mainly produced by granulosa cells of hierarchical follicles (Wang and Gong, 2017). In the largest F3-F1 preovulatory follicles, estrogen receptor mRNAs were predominantly expressed in the granular layer, suggesting that estrogen is primarily involved in regulating the granular layer through its receptors binding (Hrabia et al., 2008). Our study shows that estrogen significantly promotes the expression of the three *DHCR7* transcripts. We hypothesize that during follicle selection, estrogen acts on granulosa cells *via* paracrine binding to its receptor, enhancing *DHCR7* expression and cholesterol synthesis and providing sufficient substrate for progesterone synthesis by granulosa cells, the mechanism of which needs to be further investigated.

## 5 Conclusion

This study revealed differences in the mRNA and lncRNA transcriptomes between Pre- and Post-GCs during chicken follicle selection using Oxford Nanopore Technologies (ONT) approach and analyzed the regulation of *DHCR7* expression in chicken Pre-GCs. The data of this study may provide a reference for a better understanding of genes associated with follicle selection and theoretical basis for exploring the factors affecting the selection efficiency of chicken follicles.

## Date availability statement

The data presented in the study can be accessed from NCBI (https://www.ncbi.nlm.nih.gov/sra/docs/) with the accession number PRJNA891942.
