# Supplementary material for: Identification of genes involved in chicken follicle selection by ONT sequencing on granulosa cells
Source: Front Genet. 2023 Jan 12;13:1090603. doi: 10.3389/fgene.2022.1090603 (PMC9877231; doi:10.3389/fgene.2022.1090603)
Supplement: Supplementary file 1 [file DataSheet1.docx]

***Supplementary Material***

1. **Supplementary Figure**

**
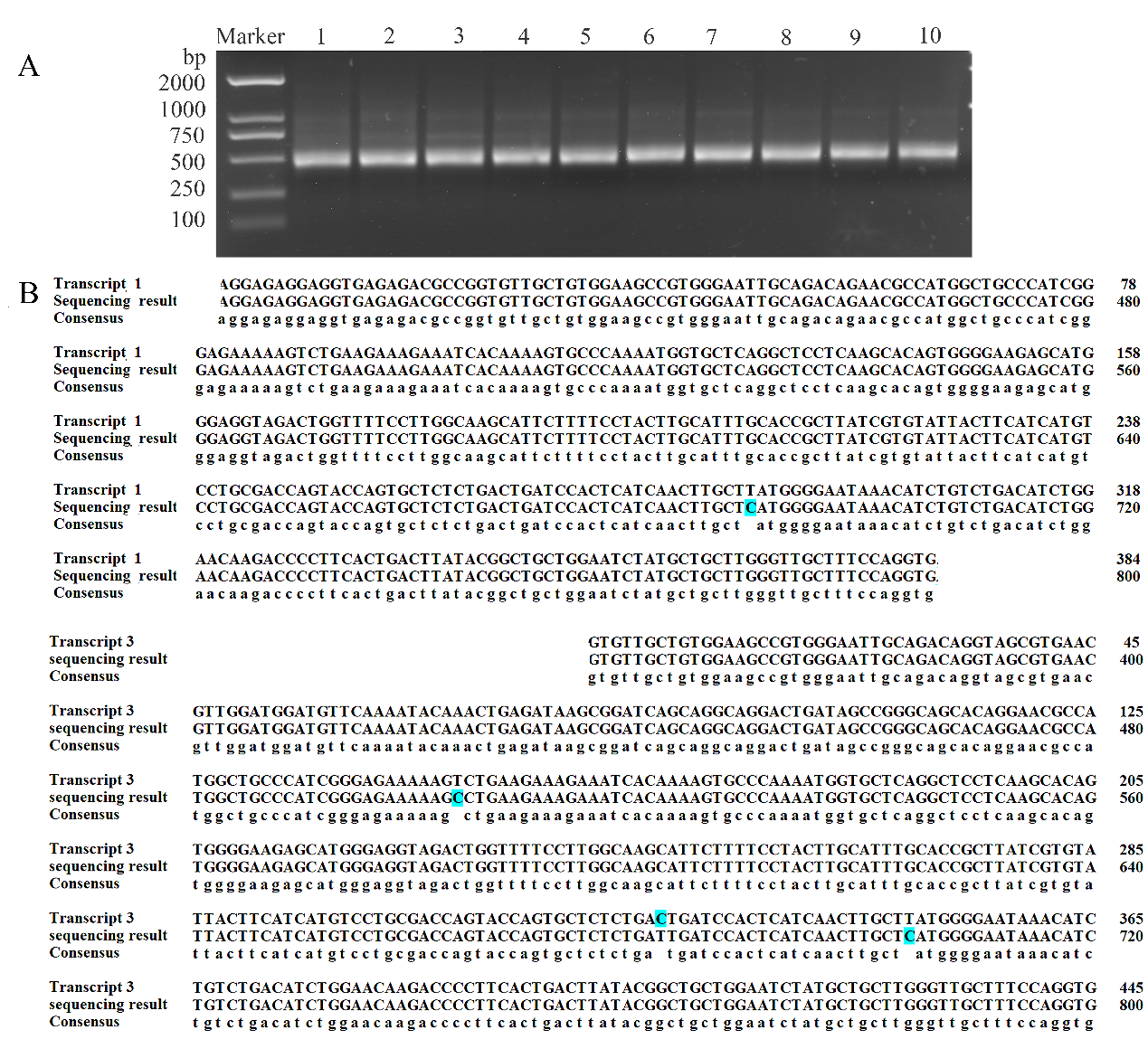
**

**Supplementary Figure 1.** Transcripts 1 and 3 of *DHCR7* are expressed in chicken granulosa cells of hierarchical follicles determined by 5' RACE. (A) Electrophoresis of 5' RACE amplification products in Post-GCs. Lanes 1 to 10 represent amplification at different annealing temperatures of 55.2, 55.5°C, 56.3°C, 57.5°C, 58.5°C, 59.5°C, 60.5°C, 61.4°C, 62.4°C, 63.5°C, 64.7°C and 65.0°C respectively (1% agarose). (B) Alignment of *DHCR7* transcript 1(upper) and transcript 3 (lower) with sequencing result by DNAMAN. The mutant nucleotide is marked by blue.

1. **Supplementary Tables**

**Supplementary Table 1.** Top 50 upregulated differentially expressed lncRNA transcripts in Post-GCs.

| **Gene ID** | **Transcript** | **Log_2_ (Foldchange)** | **padj** | **TPM**  **Pre-GCs** | **TPM**  **Post-GCs** |
| --- | --- | --- | --- | --- | --- |
| 107053110 | *LOC107053110.t4* | 6.961977789 | 4.32×10^-7^ | 0.022263333 | 5.490517333 |
| 101749492 | *LOC101749492.t4* | 6.652793548 | 6.81×10^-6^ | 0 | 1.157621667 |
| 112531969 | *LOC112531969.t2* | 6.344090427 | 9.82×10^-6^ | 0.011311667 | 1.378871333 |
| 374105 | *RIMBP2.t5* | 6.323952889 | 4.44×10^-5^ | 0 | 2.759829 |
| 414140 | *AMY2A.t4* | 6.227439855 | 5.91×10^-5^ | 0 | 1.505747667 |
| 101749492 | *LOC101749492.t2* | 6.197806095 | 2.57×10^-7^ | 0.026994 | 3.145054333 |
|  | *novel1022.t2* | 6.126736662 | 2.54×10^-5^ | 0.014599333 | 1.323167667 |
| 769469 | *PDE4D.t10* | 6.106618127 | 0.000118637 | 0 | 19.160877 |
| 417799 | *NELL2.t11* | 6.058670128 | 0.00018369 | 0 | 2.737183333 |
| 112530669 | *LOC112530669.t1* | 5.791649623 | 0.000502211 | 0 | 1.665799333 |
| 396213 | *TNR.t1* | 5.752684962 | 3.42×10^-6^ | 0.038009667 | 3.310672667 |
| 107053110 | *LOC107053110.t7* | 5.742639229 | 0.000560702 | 0 | 3.25731 |
| 769469 | *PDE4D.t2* | 5.731996597 | 0.000853767 | 0 | 2.418357 |
|  | *novel72.t1* | 5.72122407 | 0.000146623 | 0.065097333 | 5.114708333 |
| 396475 | *HAPLN1.t11* | 5.693153754 | 0.000776857 | 0 | 0.958268 |
|  | *novel178.t1* | 5.550224534 | 0.001582348 | 0 | 1.151027333 |
| 424116 | *PDE1A.t4* | 5.470218298 | 0.000404583 | 0.006095333 | 0.404740333 |
| 396475 | *HAPLN1.t10* | 5.419358467 | 0.002211711 | 0 | 1.109699333 |
| 107053720 | *LOC107053720.t2* | 5.383043097 | 0.000576084 | 0.053103 | 3.317619667 |
|  | *novel710.t1* | 5.379784374 | 0.002588028 | 0 | 0.690808333 |
| 424116 | *PDE1A.t22* | 5.375478458 | 2.88×10^-5^ | 0.022761333 | 1.322455667 |
|  | *novel298.t1* | 5.362322814 | 0.0006759 | 0.046349 | 3.815254667 |
|  | *novel32.t1* | 5.33092566 | 0.000690845 | 0.017097 | 1.407321667 |
|  | *novel326.t1* | 5.26338259 | 0.00351644 | 0 | 1.403853333 |
| 422626 | *SRP72.t1* | 5.257627138 | 0.003826975 | 0 | 0.863675667 |
|  | *novel406.t1* | 5.254525551 | 0.003750285 | 0 | 1.683504667 |
| 107053110 | *LOC107053110.t5* | 5.253550523 | 9.48×10^-18^ | 0.732643667 | 47.26435 |
| 421688 | *MAP3K5.t15* | 5.240346558 | 1.53×10^-57^ | 1.166228333 | 70.64277333 |
| 421640 | *SYNE1.t12* | 5.213435647 | 0.001144984 | 0.063804 | 4.957221667 |
| 422087 | *PGS1.t2* | 5.184590212 | 0.000137084 | 0.27367 | 14.524643 |
| 427211 | *IQGAP2.t3* | 5.170185117 | 7.75×10^-5^ | 0.014930333 | 0.821719333 |
| 770954 | *KCNK2.t1* | 5.088048228 | 0.006814264 | 0 | 4.647284667 |
| 424060 | *PLCL1.t1* | 5.072909848 | 0.001942464 | 0.105894333 | 7.198168333 |
|  | *novel529.t4* | 5.069786901 | 3.96×10^-6^ | 0.141091667 | 7.731081333 |
| 416307 | *IL9.t2* | 5.067971667 | 0.000120692 | 0.033101333 | 1.682073 |
| 421688 | *MAP3K5.t13* | 5.058512836 | 0.020405041 | 0.283981 | 12.73546767 |
| 418645 | *KIAA1324L.t6* | 5.029298084 | 0.008147298 | 0 | 0.251064 |
| 107053110 | *LOC107053110.t6* | 4.90698685 | 0.003251235 | 0.087823333 | 4.031266333 |
| 416793 | *FBRSL1.t1* | 4.889430784 | 0.017975528 | 0 | 1.789797 |
| 112531861 | *LOC112531861.t2* | 4.888167935 | 0.003723734 | 0.017839333 | 0.809392333 |
| 101750810 | *LOC101750810.t1* | 4.878893511 | 0.014285277 | 0 | 0.725135667 |
| 101748127 | *LOC101748127.t2* | 4.85799747 | 0.000340742 | 0.329405 | 17.826638 |
|  | *novel302.t1* | 4.852031347 | 0.003878285 | 0.227689 | 12.89999467 |
| 396475 | *HAPLN1.t9* | 4.831392357 | 1.37×10^-23^ | 0.253786667 | 12.084111 |
| 427113 | *MCTP1.t10* | 4.801182783 | 0.000405821 | 0.024674 | 1.098945667 |
| 420874 | *LYRM4.t5* | 4.782406208 | 0.018114517 | 0 | 0.523341333 |
| 424116 | *PDE1A.t16* | 4.754016658 | 1.03×10^-8^ | 0.083462333 | 4.109135 |
| 427754 | *UBAC1.t4* | 4.730449948 | 0.020806098 | 0 | 25.04395733 |
| 416886 | *RPH3A.t1* | 4.717486823 | 0.02025545 | 0 | 1.490843667 |
|  | *novel228.t2* | 4.716139741 | 0.006064994 | 0.033431 | 1.795465333 |

**SupplementaryTable 2.** Top 50 downregulated differentially expressed lncRNA transcripts in Post-GCs.

| **Gene ID** | **Transcript** | **Log_2_ (Foldchange)** | **padj** | **TPM**  **Pre-GCs** | **TPM**  **Post-GCs** |
| --- | --- | --- | --- | --- | --- |
| 395685 | *WSB1.t10* | -8.703881532 | 5.75×10^-11^ | 5.019506333 | 0 |
| 415797 | *CDH11.t4* | -7.771455112 | 4.27×10^-9^ | 7.024612 | 0.04414 |
| 418978 | *PDGFD.t4* | -7.477367743 | 6.55×10^-8^ | 1.829469 | 0 |
| 396210 | *NFIA.t2* | -7.160755139 | 2.83×10^-7^ | 3.302102333 | 0 |
| 395951 | *ITGA1.t3* | -7.083172365 | 4.43×10^-7^ | 1.560597 | 0 |
| 431663 | *NPR3.t2* | -6.703202667 | 3.97×10^-6^ | 3.924775 | 0 |
| 771738 | *ATOH8.t3* | -6.669604456 | 1.07×10^-8^ | 5.097679 | 0.077082 |
| 396441 | *ELN.t2* | -6.634779867 | 1.32×10^-8^ | 11.09045967 | 0.206311667 |
| 395759 | *SLC8A3.t1* | -6.613995667 | 5.59×10^-6^ | 2.693337 | 0 |
| 416099 | *PDZRN3.t6* | -6.596026816 | 8.67×10^-6^ | 1.822546333 | 0 |
| 396445 | *MYLK.t7* | -6.495221983 | 9.73×10^-6^ | 2.680757667 | 0 |
| 20744 | *BMPER.t7* | -6.433520625 | 4.63×10^-6^ | 4.776813333 | 0.076069333 |
| 396402 | *EPHA3.t4* | -6.370498193 | 1.75×10^-5^ | 1.386575 | 0 |
| 395568 | *COL5A1.t8* | -6.364321734 | 2.53×10^-38^ | 21.49964933 | 0.457103333 |
| 431603 | *ADAMTSL1.t2* | -6.33854145 | 2.38×10^-5^ | 1.954704333 | 0 |
| ADAM28 | *ADAM28.t1* | -6.293761496 | 2.84×10^-5^ | 1.883825333 | 0 |
| 419032 | *NOBOX.t1* | -6.187427408 | 1.90×10^-7^ | 2.384678 | 0.059297667 |
| 418378 | *COL8A1.t10* | -6.172057619 | 4.63×10^-5^ | 2.911054333 | 0 |
| 112530667 | *LOC112530667.t2* | -6.04867095 | 9.35×10^-5^ | 1.189474 | 0 |
| 395912 | *MGP.t1* | -5.951834214 | 9.23×10^-259^ | 753.5049847 | 19.975965 |
| 101748060 | *LOC101748060.t3* | -5.937265739 | 0.000183023 | 2.113863 | 0 |
|  | *novel1093.t2* | -5.916690753 | 5.08×10^-5^ | 11.66607667 | 0.385954333 |
| 395530 | *COL4A1.t3* | -5.842433863 | 0.000310631 | 12.116847 | 0 |
| 374173 | *SLIT3.t3* | -5.80521997 | 0.000288102 | 1.038795333 | 0 |
| 423875 | *NEURL1.t2* | -5.793840398 | 0.000290232 | 0.377483 | 0 |
| 421860 | *FILIP1.t6* | -5.675496553 | 0.000440229 | 1.05935 | 0 |
|  | *novel1026.t1* | -5.644977554 | 4.70×10^-6^ | 3.773072667 | 0.115408 |
| 396196 | *GCG.t1* | -5.594512942 | 0.000621267 | 2.996495667 | 0 |
| 426948 | *LZTS3.t2* | -5.593918958 | 0.000644967 | 2.535400667 | 0 |
| 415797 | *CDH11.t5* | -5.48887651 | 0.001025969 | 1.801728333 | 0 |
| 426948 | *LZTS3.t4* | -5.482947177 | 0.000330413 | 2.567175667 | 0.113426333 |
| 112530352 | *LOC112530352.t2* | -5.456098944 | 7.36×10^-93^ | 115.734618 | 4.263696667 |
| 426867 | *HCLS1.t1* | -5.452413544 | 0.001418564 | 2.275733333 | 0 |
|  | *novel678.t2* | -5.408203115 | 1.51×10^-5^ | 2.090610667 | 0.090814667 |
| 422771 | *GABRG1.t1* | -5.406359974 | 0.001478794 | 1.008282667 | 0 |
| 112532633 | *LOC112532633.t2* | -5.402816581 | 0.001499823 | 1.293127667 | 0 |
| 416099 | *PDZRN3.t5* | -5.3939641 | 1.71×10^-5^ | 13.395108 | 0.483228667 |
| 422413 | *FAM198B.t2* | -5.392842489 | 0.001477131 | 2.229481667 | 0 |
| 396225 | *ITGA8.t3* | -5.391989023 | 5.02×10^-22^ | 9.071526 | 0.349770333 |
| 112530775 | *LOC112530775.t1* | -5.350724659 | 0.001685671 | 2.043329667 | 0 |
| 429143 | *GPC2.t5* | -5.350626299 | 0.000495821 | 1.720484667 | 0.078993333 |
| 100858470 | *LOC100858470.t1* | -5.343409487 | 0.00163078 | 0.875324 | 0 |
| 420744 | *BMPER.t1* | -5.341939147 | 0.001663562 | 0.290971 | 0 |
| 395530 | *COL4A1.t8* | -5.333445386 | 0.000542252 | 76.45755833 | 2.700122333 |
| 386585 | *NR2F2.t4* | -5.310233901 | 1.18×10^-7^ | 15.52763867 | 0.673061333 |
| 420792 | *TNS3.t7* | -5.303449788 | 2.73×10^-5^ | 1.739694333 | 0.066424333 |
| 373965 | *CALD1.t35* | -5.294530641 | 0.002370341 | 0.460987667 | 0 |
| 107054413 | *LOC107054413.t1* | -5.292962253 | 0.002193652 | 1.165212 | 0 |
| 107052632 | *LOC107052632.t1* | -5.292190242 | 0.002015122 | 5.619827333 | 0 |
| 395813 | *HAND2.t2* | -5.252058402 | 1.58×10^-9^ | 8.251546 | 0.332037667 |

**Supplementary Table 3.** Different transcripts of the same gene displayed different expression patterns in Post-GCs.

| **GeneID** | **Transcripts** | **TPM**  **Pre-GCs** | **TPM**  **Post-GCs** | **Log_2_ (Foldchange)** | **padj** | **Regulated** |
| --- | --- | --- | --- | --- | --- | --- |
| 415697 | *AMFR.t2* | 1.82 | 1.53 | -1.025333586 | 0.021958468 | down |
|  | *AMFR.t3* | 0.00 | 1.77 | 4.540447967 | 0.037890349 | up |
| 395481 | *ANXA6.t1* | 8.70 | 33.82 | 1.257903787 | 1.03×10^-8^ | up |
|  | *ANXA6.t2* | 0.17 | 0.82 | 1.760726861 | 0.048876886 | up |
|  | *ANXA6.t3* | 0.27 | 2.34 | 2.368759982 | 1.84×10^-5^ | up |
|  | *ANXA6.t4* | 10.59 | 2.44 | -2.803185509 | 8.70052×10^-9^ | down |
| 418408 | *CD47.t2* | 2.72 | 2.26 | -1.006159463 | 0.000441742 | down |
|  | *CD47.t4* | 0.95 | 3.12 | 1.059525272 | 0.008269841 | up |
| 395274 | *FAS.t2* | 0.89 | 0.67 | -1.237995465 | 0.046836284 | down |
|  | *FAS.t4* | 1.94 | 8.02 | 1.319611879 | 8.83×10^-5^ | up |
|  | *FAS.t5* | 2.21 | 1.89 | -1.00522606 | 0.033563012 | down |
| 422970 | *GAS2.t1* | 0.44 | 2.98 | 2.021312041 | 6.17×10^-6^ | up |
|  | *GAS2.t2* | 0.24 | 2.03 | 2.248272035 | 0.047063426 | up |
|  | *GAS2.t5* | 0.89 | 0.00 | -4.285359679 | 0.044897329 | down |
| 395984 | *HDLBP.t6* | 12.37 | 6.75 | -1.640324744 | 1.55896×10^-6^ | down |
|  | *HDLBP.t8* | 0.52 | 1.70 | 1.044156619 | 0.012347595 | up |
| 396315 | *IGFBP2.t4* | 17.74 | 171.58 | 2.48601103 | 0.000202191 | up |
|  | *IGFBP2.t5* | 33.58 | 160.44 | 1.515158766 | 3.53×10^-10^ | up |
|  | *IGFBP2.t7* | 92.73 | 60.92 | -1.369513152 | 3.67276×10^-8^ | down |
|  | *IGFBP2.t8* | 1.79 | 0.74 | -2.024445283 | 0.029549009 | down |
| 417833 | *MSRB3.t1* | 0.66 | 3.50 | 1.787901469 | 0.035710632 | up |
|  | *MSRB3.t2* | 2.65 | 2.06 | -1.142396654 | 0.000276313 | down |
|  | *novel991.t1* | 2.28 | 8.70 | 1.216358022 | 0.00181717 | up |
|  | *novel991.t2* | 1.35 | 0.95 | -1.215540885 | 5.26495×10^-5^ | down |
| 422222 | *PLS3.t10* | 0.01 | 0.36 | 4.623515754 | 0.008755211 | up |
|  | *PLS3.t2* | 2.16 | 11.97 | 1.805070179 | 0.002326036 | up |
|  | *PLS3.t4* | 0.95 | 4.28 | 1.565849584 | 7.96×10^-5^ | up |
|  | *PLS3.t8* | 13.11 | 7.47 | -1.58219882 | 2.21343×10^-10^ | down |
|  | *PLS3.t9* | 0.02 | 0.99 | 5.157099125 | 9.69×10^-5^ | up |
| 427310 | *RNF20.t4* | 3.78 | 2.75 | -1.23634305 | 0.014342291 | down |
|  | *RNF20.t5* | 1.94 | 6.94 | 1.142487797 | 0.000378233 | up |
| 417637 | *RPS6KB1.t10* | 0.03 | 0.61 | 4.041299819 | 0.041646084 | up |
|  | *RPS6KB1.t12* | 1.77 | 0.66 | -2.152462634 | 0.010826448 | down |
|  | *RPS6KB1.t14* | 3.07 | 1.73 | -1.731056784 | 0.002257919 | down |
|  | *RPS6KB1.t15* | 22.86 | 15.53 | -1.272574131 | 7.76479×10^-8^ | down |
|  | *RPS6KB1.t8* | 0.96 | 9.10 | 2.550496603 | 4.03×10^-9^ | up |
|  | *RPS6KB1.t9* | 1.27 | 9.98 | 2.225756595 | 3.00×10^-10^ | up |
| 422597 | *SEC31A.t1* | 0.20 | 0.88 | 1.475486309 | 0.013500244 | up |
|  | *SEC31A.t2* | 1.26 | 0.86 | -1.31194309 | 0.000812388 | down |
| 422369 | *SEPT6.t2* | 0.44 | 1.68 | 1.310061574 | 0.029341747 | up |
|  | *SEPT6.t4* | 1.88 | 0.53 | -2.552690582 | 9.27673×10^-6^ | down |
| 420896 | *SERPINB2.t2* | 2.93 | 45.03 | 3.230246163 | 3.81×10^-58^ | up |
|  | *SERPINB2.t3* | 0.11 | 1.67 | 2.908790134 | 0.004751839 | up |
|  | *SERPINB2.t5* | 189.82 | 36.24 | -3.199229504 | 0.018447635 | down |
| 100858894 | *SMTN.t1* | 0.84 | 4.28 | 1.665709228 | 0.006558025 | up |
|  | *SMTN.t5* | 0.93 | 0.22 | -2.774970382 | 0.011543052 | down |

**Supplementary Table 4.** Differentially expressed genes related to hormone activity, cell differentiation, cholesterol biosynthetic process, steroid hormone receptor activity and response to cAMP.

| **Classification** | **GeneID** | **GeneName** | **Log_2_ (Foldchange)** | **Padj** | **Regulated** |
| --- | --- | --- | --- | --- | --- |
| hormone activity | 428201 | *STC1* | 2.514459467 | 1.0095×10^-111^ | up |
|  | 427223 | *RLN3* | 9.739726137 | 1.69435×10^-69^ | up |
|  | 420854 | *EDN1* | 2.126524178 | 2.86×10^-44^ | up |
|  | 396277 | *TTR* | -2.276275674 | 5.08368×10^-27^ | down |
|  | 420716 | *LOC420716* | -2.198797913 | 5.09418×10^-21^ | down |
|  | 373992 | *FBN1* | -2.387518289 | 9.47807×10^-20^ | down |
|  | 417332 | *METRNL* | -1.216636917 | 1.35×10^-13^ | down |
|  | 424197 | *INHA* | 6.791500761 | 1.81×10^-09^ | up |
|  | 419422 | *FAM132A* | -5.619470075 | 2.53×10^-9^ | down |
|  | 419667 | *FNDC5* | -2.63550021 | 2.37×10^-7^ | down |
|  | 396281 | *PTHLH* | 3.193294918 | 7.78×10^-7^ | up |
|  | 112532887 | *ADM2* | -2.855147465 | 0.001206631 | down |
| cell differentiation | 395912 | *MGP* | -5.950634075 | 0 | down |
|  | 386585 | *NR2F2* | -4.630780727 | 2.87485×10^-64^ | down |
|  | 374100 | *FLT1* | 3.237182979 | 1.39×10^-46^ | up |
|  | 423196 | *MDK* | -5.970665269 | 5.28408×10^-45^ | down |
|  | 420741 | *SEPT7* | -2.240944738 | 1.31819×10^-38^ | down |
|  | 770787 | *FHL1* | -6.402614178 | 1.28082×10^-37^ | down |
|  | 770845 | *NELL1* | 3.833290214 | 1.02117×10^-28^ | up |
|  | 422982 | *DHCR7* | 2.007675005 | 2.32×10^-26^ | up |
|  | 396104 | *SOX11* | -3.41489122 | 2.40927×10^-24^ | down |
|  | 395349 | *FSTL1* | -1.677713762 | 6.52372×10^-22^ | down |
|  | 100859853 | *GADD45B* | -1.932397871 | 8.64359×10^-21^ | down |
|  | 429806 | *HSPG2* | 3.202771126 | 3.78176×10^-20^ | down |
|  | 771307 | *FAM129B* | -2.459075534 | 4.47×10^-20^ | down |
|  | 419245 | *PPDPF* | -1.558925493 | 9.98×10^-17^ | down |
|  | 771113 | *TESC* | -1.920823269 | 8.05×10^-13^ | down |
|  | 395670 | *MEF2A* | -1.903428834 | 6.23×10^-12^ | down |
|  | 771738 | *ATOH8* | -6.236891544 | 1.49624×10^-11^ | down |
|  | 373917 | *LGALS3* | -1.466904673 | 2.89×10^-11^ | down |
|  | 374146 | *ERG* | -2.3315666 | 3.75901×10^-10^ | down |
|  | 395610 | *ANGPT2* | -1.972879807 | 1.8461×10^-9^ | down |
|  | 395879 | *SPI1* | -7.816905272 | 2.14×10^-9^ | down |
|  | 425859 | *EXT2* | -1.342037013 | 3.44×10^-9^ | down |
|  | 424367 | *HSD17B7* | 1.478769577 | 2.22687×10^-8^ | up |
|  | 415706 | *OSGIN1* | -3.933347007 | 1.46×10^-7^ | down |
|  | 395241 | *HOPX* | -6.024716076 | 1.83575×10^-7^ | down |
|  | 417837 | *CAND1* | 1.004350482 | 1.40×10^-6^ | up |
|  | 395887 | *AMH* | -3.285132451 | 2.02×10^-6^ | down |
|  | 422593 | *ARHGAP24* | 1.343714356 | 2.44612×10^-6^ | up |
|  | 417768 | *ASZ1* | 6.513322617 | 4.25×10^-6^ | up |
|  | 423477 | *TNFAIP2* | -1.840081971 | 6.65857×10^-6^ | down |
|  | 374024 | *TWSG1* | -1.383488247 | 8.46×10^-6^ | down |
|  | 374120 | *PPARA* | 1.444252136 | 4.57×10^-5^ | up |
|  | 396413 | *FGF2* | -3.280579082 | 5.21×10^-5^ | down |
|  | 395466 | *CSPG5* | 3.520251022 | 0.00011681 | up |
|  | 423737 | *CAMK2G* | -1.76800231 | 0.000121659 | down |
|  | 770683 | *HIP1* | -1.902740845 | 0.00020028 | down |
|  | 427947 | *PTPN6* | -5.537025347 | 0.000304373 | down |
|  | 396404 | *DDR2* | -1.337302212 | 0.00060359 | down |
|  | 417619 | *NXN* | 5.041860619 | 0.000874344 | up |
|  | 426314 | *SUV39H2* | 1.113217807 | 0.000887096 | up |
|  | 395747 | *ETV4* | -1.332237288 | 0.002048089 | down |
|  | 424506 | *BRDT* | 1.648464244 | 0.003198515 | up |
|  | 427378 | *HEMGN* | 1.848966419 | 0.004878036 | up |
|  | 421127 | *LYN* | -1.631266239 | 0.004933405 | down |
|  | 395441 | *TLL1* | -2.737631636 | 0.004939916 | down |
|  | 395546 | *SFRP2* | 1.653359151 | 0.006121033 | up |
|  | 395655 | *NOTCH1* | -1.230485445 | 0.006635389 | down |
|  | 101749628 | *LOC101749628* | -4.75997396 | 0.00691522 | down |
|  | 419929 | *TAF8* | 1.289761175 | 0.007081759 | up |
|  | 417460 | *FKBP6* | -3.767766129 | 0.007777092 | down |
|  | 422035 | *BLK* | -4.502934014 | 0.011607885 | down |
|  | 395750 | *ETV6* | -1.37834228 | 0.015266815 | down |
|  | 431601 | *UHRF2* | -1.722083833 | 0.016142032 | down |
|  | 424363 | *HTATIP2* | -1.717942748 | 0.026061776 | down |
|  | 100859273 | *LOC100859273* | -1.099339527 | 0.030704876 | down |
|  | 427201 | *ARHGEF28* | -3.223722922 | 0.032137631 | down |
|  | 423285 | *EIF2AK4* | -1.17811636 | 0.048412411 | down |
| cholesterol biosynthetic process | 396379 | *HMGCS1* | 3.871509448 | 5.18×10^-220^ | up |
|  | 396536 | *APOA1* | -2.519049776 | 1.88×10^-87^ | down |
|  | 424661 | *DHCR24* | -4.081698019 | 3.63×10^-82^ | up |
|  | 422038 | *FDFT1* | 2.130886005 | 3.64×10^-38^ | up |
|  | 422982 | *DHCR7* | 2.007675005 | 2.32×10^-26^ | up |
|  | 424276 | *INSIG2* | -3.786489375 | 1.06×10^-11^ | down |
|  | 420459 | *IDI1* | -1.137598778 | 1.95×10^-11^ | up |
|  | 395373 | *ACLY* | 1.225071568 | 1.36×10^-8^ | up |
|  | 424367 | *HSD17B7* | 1.478769577 | 2.23×10^-8^ | up |
|  | 422302 | *NSDHL* | 1.587536938 | 9.86×10^-8^ | up |
|  | 425359 | *MVD* | 2.182305393 | 0.000104674 | up |
|  | 420442 | *INSIG1* | -1.051398898 | 0.00746059 | down |
| steroid hormone receptor activity | 386585 | *NR2F2* | -4.630780727 | 2.87×10^-64^ | down |
|  | 395960 | *NR5A1* | 3.792569466 | 9.51×10^-22^ | up |
|  | 415493 | *ABHD2* | 1.286073565 | 2.51×10^-12^ | up |
|  | 425505 | *PAQR7* | 1.178260853 | 8.90×10^-6^ | up |
|  | 771938 | *ESRRB* | 2.170041715 | 0.001814837 | up |
|  | 395988 | *VDR* | 1.409896688 | 0.045291102 | up |
| response to cAMP | 421960 | *SDC1* | 1.599252502 | 8.64×10^-43^ | up |
|  | 427391 | *CDO1* | 3.78863873 | 2.63×10^-17^ | up |
|  | 427381 | *DGKQ* | 2.730461015 | 1.05×10^-16^ | up |
|  | 107055370 | *JUND* | -1.482714934 | 0.044139216 | down |

**Supplementary Table 5.** Top 50 upregulated differentially expressed genes in Post-GCs.

| **GeneID** | **GeneName** | **Log_2_ (Foldchange)** | **Padj** | **TPM**  **Pre-GCs** | **TPM**  **Post-GCs** |
| --- | --- | --- | --- | --- | --- |
| 378906 | *ZP3* | 6.063012389 | 0 | 59.56 | 6636.06 |
| 396015 | *HSD3B1* | 4.820070595 | 1.28×10^-302^ | 4.93 | 221.55 |
| 417967 | *PLBD1* | 4.595109555 | 3.00×10^-271^ | 3.07 | 89.62 |
| 421604 | *MPPED2* | 4.422343726 | 4.17×10^-244^ | 48.56 | 322.77 |
| 427449 | *DAB2* | 4.066293314 | 4.57×10^-224^ | 9.31 | 362.39 |
| 768821 | *FGL2* | 5.127477455 | 5.53×10^-224^ | 6.19 | 134.63 |
| 396379 | *HMGCS1* | 3.871509448 | 5.18×10-^220^ | 3.36 | 81.81 |
| 112532135 | *LOC112532135* | 3.613104487 | 3.16×10^-204^ | 19.14 | 378.84 |
|  | *novel75* | 3.333038871 | 2.68×10^-202^ | 10.48 | 168.30 |
|  | *novel346* | 3.644547773 | 2.09×10^-195^ | 17.04 | 351.61 |
| 423274 | *RDH12* | 3.639887279 | 8.62×10^-173^ | 4.09 | 85.29 |
| 423272 | *ARG2* | 4.063680589 | 3.90×10^-170^ | 5.78 | 164.27 |
| 423353 | *NPC2* | 3.027588289 | 2.87×10^-166^ | 29.70 | 403.35 |
| 416308 | *SLC25A48* | 5.432990992 | 6.73×10^-156^ | 0.37 | 27.80 |
|  | *novel73* | 3.909415229 | 8.47×10^-154^ | 27.91 | 652.41 |
|  | *novel527* | 4.789992002 | 5.68×10^-148^ | 3.02 | 128.61 |
| 112532134 | *LOC112532134* | 2.875077314 | 2.63×10^-140^ | 52.83 | 643.01 |
| 428163 | *CTSA* | 3.083803378 | 5.71×10^-135^ | 16.07 | 223.30 |
| 420384 | *AQP1* | 3.498394949 | 8.94×10^-133^ | 3.93 | 72.85 |
|  | *novel226* | 2.8033565 | 5.08×10^-129^ | 18.78 | 218.34 |
| 427211 | *IQGAP2* | 3.281662837 | 2.08×10^-128^ | 9.10 | 111.22 |
| 427284 | *CLTA* | 2.973015147 | 4.00×10-^123^ | 29.69 | 391.58 |
| 418549 | *PDXK* | 2.671467781 | 1.19×10^-118^ | 5.76 | 104.09 |
| 396530 | *ATP1A1* | 2.630046629 | 2.56×10^-117^ | 20.03 | 100.23 |
| 552895 | *ALAS1* | 2.946581223 | 3.17×10^-115^ | 24.93 | 244.29 |
| 422172 | *FDX1L* | 3.430385165 | 2.11×10^-113^ | 2.28 | 41.57 |
| 428201 | *STC1* | 2.514459467 | 1.01×10^-111^ | 25.97 | 305.03 |
| 107052389 | *LOC107052389* | 2.665318643 | 5.89×10^-109^ | 1657.06 | 8545.35 |
| 404754 | *UMOD* | 4.763149414 | 3.50×10^-106^ | 1.54 | 68.80 |
| 395939 | *DIO3* | 4.217421721 | 2.94×10^-105^ | 2.14 | 65.97 |
|  | *novel184* | 2.470547971 | 3.40×10^-101^ | 96.79 | 919.46 |
| 418981 | *MMP10* | 4.889259309 | 4.83×10^-101^ | 1.07 | 54.55 |
| 378916 | *LHFPL5* | 3.651114108 | 5.95×10^-101^ | 4.11 | 86.03 |
|  | *novel1022* | 3.194851869 | 1.00×10^-99^ | 1.72 | 26.44 |
|  | *novel41* | 3.620555121 | 6.41×10^-97^ | 1.95 | 43.82 |
| 428164 | *PLTP* | 3.301599674 | 2.33×10^-96^ | 4.90 | 79.75 |
| 427978 | *MRPS6* | 2.650918155 | 1.22×10^-94^ | 9.96 | 101.20 |
| 112531968 | *LOC112531968* | 3.630285509 | 8.28×10^-92^ | 8.19 | 164.33 |
| 768420 | *COTL1* | 2.204263759 | 1.27×10^-90^ | 30.17 | 230.36 |
| 422087 | *PGS1* | 2.894403596 | 1.56×10^-88^ | 4.84 | 61.41 |
| 417799 | *NELL2* | 2.911778335 | 1.14×10^-87^ | 4.89 | 489.48 |
| 424661 | *DHCR24* | 4.081698019 | 3.63×10^-82^ | 0.55 | 15.73 |
| 419528 | *NEFL* | 4.599426223 | 1.11×10^-80^ | 0.69 | 27.97 |
| 416371 | *BHMT* | 3.521892476 | 4.63×10^-79^ | 3.52 | 67.37 |
| 424910 | *CLDN1* | 3.274272576 | 7.95×10^-79^ | 2.26 | 33.90 |
| 100858006 | *SELENOU* | 2.8597252 | 6.66×10^-78^ | 7.26 | 81.83 |
| 424810 | *ACSL3* | 2.430442438 | 1.63×10^-77^ | 22.36 | 253.11 |
| 426456 | *LOC426456* | 3.981694097 | 1.14×10^-76^ | 0.55 | 14.51 |
| 418265 | *MAN1A2* | 4.193702018 | 2.40×10^-76^ | 68.57 | 888.12 |
|  | *novel529* | 4.200646273 | 1.10×10^-74^ | 0.46 | 15.67 |

**Supplementary Table 6.** Top 50 downregulated differentially expressed genes in Post-GCs.

| **GeneID** | **GeneName** | **Log_2_ (Foldchange)** | **padj** | **TPM**  **Pre-GCs** | **TPM**  **Post-GCs** |
| --- | --- | --- | --- | --- | --- |
| 395912 | *MGP* | -5.950634075 | 0 | 753.51 | 19.98 |
| 373965 | *CALD1* | -5.14174875 | 1.74×10^-273^ | 448.07 | 19.74 |
| 396243 | *COL1A2* | -4.581985932 | 1.09×10^-248^ | 89.61 | 4.71 |
| 396490 | *TAGLN* | -4.531366887 | 6.77×10^-214^ | 275.69 | 19.76 |
| 396340 | *COL3A1* | -3.428212928 | 3.98×10^-210^ | 262.18 | 59.44 |
| 396491 | *LGALS1* | -5.439318769 | 1.48×10^-200^ | 459.39 | 17.27 |
| 396465 | *MYH10* | -3.867804746 | 5.38×10^-173^ | 78.71 | 9.34 |
| 396445 | *MYLK* | -4.987594804 | 3.95×10^-170^ | 59.07 | 2.52 |
| 395527 | *CD9* | -3.770670188 | 4.19×10^-148^ | 36.41 | 4.46 |
| 396366 | *TPM1* | -2.531489972 | 4.23×10^-123^ | 553.47 | 145.21 |
| 395532 | *COL1A1* | -6.624218482 | 1.19×10^-118^ | 287.60 | 3.55 |
| 112530352 | *LOC112530352* | -5.523232461 | 5.21×10^-111^ | 539.89 | 4.26 |
| 395568 | *COL5A1* | -6.297332902 | 1.28×10^-109^ | 196.28 | 1.08 |
| 395772 | *KRT7* | -3.140836028 | 3.10×10^-109^ | 53.78 | 8.56 |
| 396084 | *ACTG2* | -3.449208954 | 1.82×10^-103^ | 168.54 | 25.66 |
| 417892 | *DCN* | -6.03536094 | 7.10×10^-95^ | 34.57 | 0.69 |
| 422810 | *SOD3* | -3.408131181 | 7.10×10^-93^ | 92.31 | 14.25 |
| 396536 | *APOA1* | -2.519049776 | 1.88×10^-87^ | 952.34 | 272.34 |
| 421534 | *ACTA1* | -4.464754516 | 5.17×10^-87^ | 35.39 | 2.62 |
| 395657 | *SPON1* | -2.733322736 | 6.52×10^-87^ | 1069.72 | 120.79 |
| 100859797 | *ACKR4* | -4.247176503 | 1.07×10^-81^ | 21.71 | 1.95 |
| 395550 | *LY6E* | -3.76797266 | 2.97×10^-81^ | 13.72 | 1.62 |
| 423986 | *COL5A2* | -4.255413981 | 6.91×10^-76^ | 13.89 | 1.29 |
| 386583 | *MMP2* | -4.111446125 | 3.09×10^-67^ | 12.97 | 1.00 |
| 404271 | *ANXA1* | -3.7964932 | 1.01×10^-66^ | 25.05 | 2.95 |
| 768390 | *PDPN* | -4.315418291 | 5.94×10^-66^ | 15.73 | 1.34 |
| 396469 | *MYH9* | -1.771306492 | 1.20×10^-65^ | 78.39 | 62.65 |
| 386585 | *NR2F2* | -4.630780727 | 2.87×10^-64^ | 75.57 | 5.13 |
| 374062 | *FZD1* | -3.118434507 | 1.12×10^-63^ | 58.26 | 14.05 |
| 423504 | *HSPA2* | -2.480409996 | 6.49×10^-63^ | 281.84 | 75.74 |
| 423789 | *LIPA* | -3.115919149 | 6.79×10^-63^ | 41.90 | 2.50 |
| 396506 | *S100A10* | -1.964697494 | 1.21×10^-61^ | 1232.50 | 529.67 |
| 418378 | *COL8A1* | -5.011854484 | 1.39×10^-61^ | 161.10 | 12.44 |
| 396432 | *TGM2* | -3.69578029 | 1.47×10^-57^ | 10.18 | 1.32 |
| 395074 | *CCDC80* | -2.412799276 | 1.93×10^-54^ | 66.98 | 7.83 |
| 418752 | *COL4A2* | -5.530184883 | 5.62×10^-54^ | 15.06 | 0.90 |
| 395970 | *FTH1* | -1.735145541 | 7.07×10^-54^ | 1181.16 | 585.53 |
| 396246 | *FABP7* | -2.445409737 | 6.37×10^-52^ | 122.69 | 35.71 |
| 428543 | *EFEMP1* | -2.682582081 | 7.94×10^-51^ | 45.65 | 4.47 |
| 374254 | *LSP1* | -5.323970552 | 8.25×10^-50^ | 13.91 | 0.60 |
| 396297 | *ANXA2* | -2.061077583 | 3.10×10^-49^ | 96.27 | 37.69 |
| 374157 | *NRP2* | -4.450767571 | 3.83×10^-48^ | 605.36 | 36.49 |
| 396090 | *CTSD* | -2.17818948 | 1.48×10^-47^ | 16.79 | 6.28 |
| 776594 | *LOC776594* | -3.714929935 | 3.78×10^-47^ | 17.31 | 2.25 |
| 420129 | *IFI30* | -3.739495847 | 1.24×10^-46^ | 31.17 | 3.82 |
| 428437 | *CHN2* | -4.348644403 | 2.37×10^-45^ | 17.77 | 1.52 |
| 423196 | *MDK* | -5.970665269 | 5.28×10^-45^ | 72.75 | 1.29 |
| 424882 | *PLOD2* | -2.396501199 | 5.26×10^-44^ | 17.90 | 9.42 |
| 395818 | *CTSK* | -6.519042948 | 9.61×10^-44^ | 31.37 | 0.59 |
| 373979 | *FBLN1* | -2.317060399 | 1.04×10^-43^ | 41.71 | 14.40 |
